# Conversion-type anode chemistry with interfacial compatibility toward Ah-level near-neutral high-voltage zinc ion batteries

**DOI:** 10.1093/nsr/nwae181

**Published:** 2024-05-25

**Authors:** Shan Guo, Liping Qin, Jia Wu, Zhexuan Liu, Yuhao Huang, Yiman Xie, Guozhao Fang, Shuquan Liang

**Affiliations:** School of Materials Science and Engineering, Key Laboratory of Electronic Packaging and Advanced Functional Materials of Hunan Province, Central South University, Changsha 410083, China; College of Biological and Chemical Engineering, Guangxi University of Science and Technology, Liuzhou 545006, China; School of Materials Science and Engineering, Key Laboratory of Electronic Packaging and Advanced Functional Materials of Hunan Province, Central South University, Changsha 410083, China; School of Materials Science and Engineering, Key Laboratory of Electronic Packaging and Advanced Functional Materials of Hunan Province, Central South University, Changsha 410083, China; School of Materials Science and Engineering, Key Laboratory of Electronic Packaging and Advanced Functional Materials of Hunan Province, Central South University, Changsha 410083, China; Information and Network Center, Central South University, Changsha 410083, China; School of Materials Science and Engineering, Key Laboratory of Electronic Packaging and Advanced Functional Materials of Hunan Province, Central South University, Changsha 410083, China; School of Materials Science and Engineering, Key Laboratory of Electronic Packaging and Advanced Functional Materials of Hunan Province, Central South University, Changsha 410083, China

**Keywords:** zinc compound anode, conversion reaction, voltage regulation, interfacial design, pouch cell

## Abstract

High-voltage aqueous zinc ion batteries (AZIBs) with a high-safety near-neutral electrolyte is of great significance for practical sustainable application; however, they suffer from anode and electrode/electrolyte interfacial incompatibility. Herein, a conversion-type anode chemistry with a low anodic potential, which is guided by the Gibbs free energy change of conversion reaction, was designed for high-voltage near-neutral AZIBs. A reversible conversion reaction between ZnC_2_O_4_·2H_2_O particles and three-dimensional Zn metal networks well-matched in CH_3_COOLi-based electrolyte was revealed. This mechanism can be universally validated in the battery systems with sodium or iodine ions. More importantly, a cathodic crowded micellar electrolyte with a water confinement effect was proposed in which lies the core for the stability and reversibility of the cathode under an operating platform voltage beyond 2.0 V, obtaining a capacity retention of 95% after 100 cycles. Remarkably, the scientific and technological challenges from the coin cell to Ah-scale battery, sluggish kinetics of the solid-solid electrode reaction, capacity excitation under high loading of active material, and preparation complexities associated with large-area quasi-solid electrolytes, were explored, successfully achieving an 88% capacity retention under high loading of more than 20 mg cm^−2^ and particularly a practical 1.1 Ah-level pouch cell. This work provides a path for designing low-cost, eco-friendly and high-voltage aqueous batteries.

## Teaser

This paper proposes the conversion-type anode chemistry and interfacial compatibility to design Ah-level near-neutral high-voltage zinc ion batteries.

## INTRODUCTION

Development of sustainable energy is in urgent need of high safety, low cost, and green electrochemical energy storage systems. Aqueous zinc ion batteries (AZIBs) can well meet these demands due to the adoption of a zinc metal (Zn) anode and near-neutral aqueous electrolyte [1–3]. However, they come at the cost of sacrificing operating voltage (all below 2.0 V *vs.* Zn^2+^/Zn, as seen in Fig. S1) [4–7], which greatly limits their energy density output. It was reported that the operating voltage is often elevated by ion-exchange membranes and strong acid-base electrolyte [8]; unfortunately, this is a neglected field when designing novel electrode couples in near-neutral aqueous electrolyte.

At present, the mainstream anodes for AZIBs are Zn anodes with the electrode potential of −0.76 V (*vs*. standard hydrogen electrode (SHE)) [9–12], which are impracticable to match with the cathode materials to achieve high platform voltage in near-neutral electrolyte [13,14]. In addition, dendrites, corrosion, hydrogen evolution, etc., seriously challenge the practicability of Zn anodes [15–19]. Some embedded Zn compounds such as Ti_2_O(PO_4_)_2_·2H_2_O [20], Cu_7_Te_4_ [21], etc. suffer from high anodic potential compared to that of Zn anodes, which is even less able to attain high platform voltage. The conversion-type anode based on a Zn/Zn^2+^-compound pair with a large Gibbs free energy change is expected to reduce the anodic potential (Fig. S2), but there have been no reports of this at present. Recently, a solid-to-solid conversion electrochemistry (e.g. ZnCO_3_·3Zn(OH)_2_) for anode has been proposed [22]; however, it is limited by the alkaline ZIBs. Fixation of the electrolyte will subsequently lead to a limited choice of cathodes, hindering the exploration of high-voltage near-neutral AZIBs. The conversion-type anode, which does not depend on a specific battery system, is an effective way to solve this difficulty, but it lacks conceptual design.

Another common challenge of securing high-voltage near-neutral AZIBs stems from the electrolyte/electrode interfacial compatibility [23–25], due to the intrinsic narrow electrochemical stability of aqueous electrolyte [5,26,27]. High-voltage operating conditions will trigger the oxygen evolution reaction and structural degradation of cathode materials, leading to a shorter cycle life [28,29]. To broaden the electrochemical stability of aqueous electrolyte, ‘salt-in-water’ electrolyte [30], eutectic electrolyte [31], molecular crowding electrolytes [32], etc. have been proposed, which provided strong evidence for developing high-voltage AZIBs due to the effective reduction of water activity in the electrolyte. However, the use of highly concentrated bis(trifluoromethane)sulfonimide salts (e.g. LiTFSI) or large amounts of organic component leads to cost, toxicity, or safety concerns. More importantly, unidirectional regulation of electrolyte for cathode/electrolyte interface is mostly unsuitable for the anodic Zn/Zn^2+^-compound conversion reaction, because of the different mechanisms and shortcomings of cathode and anode [33–35]. Therefore, how to adjust the electrolyte to be compatible with both electrodes lies at the heart of the stable operation of high-voltage near-neutral AZIBs.

Herein, we propose a conversion-type anode chemistry toward high-voltage near-neutral AZIBs, which is universally validated in diverse battery systems. The Gibbs free energy change, governed by the Zn/Zn^2+^-compound conversion reaction, directs the selection of a low-potential ZnC_2_O_4_·2H_2_O anode. A reversible transformation between ZnC_2_O_4_·2H_2_O particles and three-dimensional Zn metal networks well-matched by CH_3_COOLi-based electrolyte was revealed. A crowded micellar electrolyte with a water confinement effect was proposed to distinguish the interface difference between the anode and cathode, which enables an operating platform voltage beyond 2.0 V for near-neutral AZIBs by suppressing electrolyte decomposition and cathode particle cracking. Based on this design, the battery could maintain >100 cycles with a capacity retention of 95%. Importantly, the practical and stable Ah-scale pouch batteries were successfully achieved and provided scientific and technical guidance for practical applications, which is often ignored in most of previous works.

## RESULTS AND DISCUSSION

### Low potential conversion-type anode and regulation mechanism

**Figure 1.**
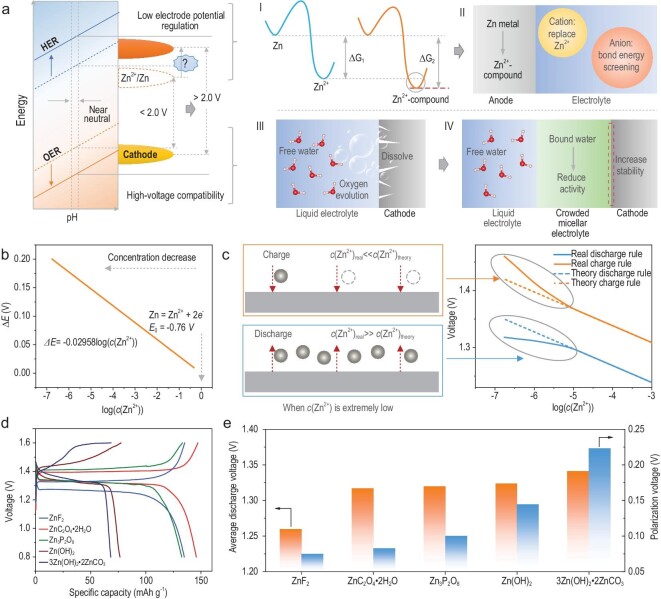
The regulation of high-voltage aqueous zinc ion batteries guided by the anodic electrode potential. (a) Design principles of high-voltage aqueous zinc ion batteries, in terms of a Zn/Zn^2+^-compound conversion anode (inset **I**), the compatible electrolyte with anode (inset **II**), and the compatibility of the high-voltage cathodic interface (inset **III and IV**). (b) Theoretical relationship between *c*(Zn^2+^) and changes of anode electrode potential. (c) Real relationship between *c*(Zn^2+^) and changes of anode electrode potential when *c*(Zn^2+^) is extremely low. (d) The effect of different Zn^2+^-compound anodes on the GCD curve of LiFePO_4_. (e) The relationship between average discharge voltage, polarization voltage, and types of Zn^2+^-compound anodes.

Regulating the electrode potential of the anode, beyond that of conventional zinc metal anode, set the primary criteria for high-voltage AZIB battery systems. Figure S2 illustrates the relationship between the Gibbs free energy change (∆G) and the anodic electrode potential. A Zn/Zn^2+^-compound conversion reaction with large ∆G will possess lower electrode potential than that of Zn/Zn^2+^ reaction and intercalative anode/Zn^2+^ reaction (inset **I** of Fig. 1a). For the Zn^2+^-compound anode, the anions with stronger binding ability (such as C_2_O_4_^2−^ or CO_3_^2−^) will bind to the stripped Zn^2+^ to produce a Zn^2+^-compound precipitation (ZnC_2_O_4_·2H_2_O, 3Zn(OH)_2_·2ZnCO_3_, etc.) at oxidation state, resulting in larger ∆G compared to that from Zn metal to free state Zn^2+^ (Fig. S3). Therefore, the electrode potential of Zn^2+^-compound anode is generally lower than that of the standard Zn anode, which encouraged us to explore the feasibility of this new type of anode.

Zn^2+^-compounds such as ZnC_2_O_4_·2H_2_O, Zn_3_(C_6_H_5_O_7_)_2_·2H_2_O, 3Zn(OH)_2_·2ZnCO_3_, Zn(OH)_2_, ZnF_2_, Zn_3_P_2_O_8_, etc., which are slightly soluble in aqueous electrolyte, are the range to be considered. To explore the regulation mechanism of electrode potential of the conversion-type anode, LiFePO_4_ was chosen as the cathode due to its moderate electrode potential, which can eliminate the effect of high voltage on the cathode side. After a series of electrolyte controls, the plateau voltage rises noticeably in the battery with conversion-type anode (e.g. ZnC_2_O_4_·2H_2_O), which was 0.15 V higher than that of the common system (Fig. S4). During the reduction process the Zn^2+^-compound is reduced to zinc metal and anion groups; conversely, during the oxidation process Zn^2+^ binds the anion groups to reproduce the Zn^2+^-compound precipitation, thus reducing the Zn^2+^ concentration in the interface. However, it should be noted that the normal operation of the Zn/Zn^2+^-compound conversion reaction depends on the specific electrode/electrolyte interfacial reaction (inset **II** of Fig. 1a). The binding energy of anions in the anodic electrolyte needs to be lowed to prevent the competitive reaction between anions with Zn^2+^-compounds (Fig. S5). The detailed conversion mechanism will be discussed in depth later.

The decrease of Zn^2+^ concentration increases the plateau voltage which can be analyzed by Nernst equation. According to the Nernst equation of the electrode potential (Equation 1):


(1) \begin{equation*}E = {{E}_0} + \frac{{RT}}{{nF}}\ln \frac{{c\left( {{\mathrm{oxidation\ state}}} \right)}}{{c\left( {{\mathrm{reduction\ state}}} \right)}},\end{equation*}

where *E* is the electrode potential; ${{E}_0}$ is the electrode potential at standard state; *R* is gas constant; *T* is temperature; *n* is number of electrons transferred in electrode reaction; *F* is Faraday’s constant; $c( {{\mathrm{oxidation\ state}}} )$ and $c( {{\mathrm{reduction\ state}}} )$ represent the concentrations of the oxidation and reduction states, respectively. The relationship between electrode potential and concentration of Zn^2+^ (*c*(Zn^2+^)) could be obtained (Equation 2) based on the reaction of Zn anode.


(2) \begin{equation*}E = {{E}_0} + \frac{{0.05916}}{2}\log c\left( {{\mathrm{Z}}{{{\mathrm{n}}}^{2 + }}} \right)\end{equation*}

Using ${\vartriangle} E$ to represent the difference between the electrode potential at standard state and the electrode potential (${{E}_0} - E$), the above formula can be further converted to Equation 3.


(3) \begin{equation*}\Delta E = - 0.02958\log c\left( {{\mathrm{Z}}{{{\mathrm{n}}}^{2 + }}} \right)\end{equation*}

$\Delta E$ also represents the voltage increase of the battery system. The relationship between ${\vartriangle} E$ and *c*(Zn^2+^) is graphically illustrated in Fig. 1b. It can be seen that $\Delta E$ increases when the *c*(Zn^2+^) decreases. Compared with Zn^2+^ solution, the Zn^2+^ concentration from the decomposition of ZnC_2_O_4_·2H_2_O is significantly lower, thus there is a large increase in platform voltage. However, the extremely low *c*(Zn^2+^) may lead to the shortage of reactants during Zn^2+^ deposition, thereby limiting the interfacial reaction. When *c*(Zn^2+^) is extremely low, the *c*(Zn^2+^) is susceptible to significant fluctuations during the charge/discharge process. In the charge stage, the consumption of Zn^2+^ will lead to the real *c*(Zn^2+^) which is far less than the theoretical *c*(Zn^2+^), while the discharge process is the opposite (Fig. 1c). According to Equation 3, the above phenomena will lead to the real charge voltage being higher than the theoretical value, while the real discharge voltage is lower than the theoretical value. Therefore, the extremely low *c*(Zn^2+^) will result in a rapid increase in the polarization voltage. Figure S6 vividly summarizes the reduction of *c*(Zn^2+^) on the influence of the comprehensive electrochemical performance. When the Zn^2+^ are sufficient, the reduction of *c*(Zn^2+^) can quickly improve operating voltage. However, when Zn^2+^ are deficient, the effect of voltage increase gradually weakens instead, and the dynamic behavior becomes unfavorable. Based on the above analysis, there is an optimal concentration to achieve comprehensive performance improvement.

To confirm the above analysis, the Zn^2+^-compounds with similar electronic structure but different solubility products, including ZnF_2_, ZnC_2_O_4_·2H_2_O, Zn_3_P_2_O_8_, Zn(OH)_2_ and 3Zn(OH)_2_·2ZnCO_3_ have been selected for testing. As shown in Fig. 1d, the main phenomenon is that the ZnF_2_ anode only has a discharge platform voltage of ∼1.25 V, while Zn(OH)_2_ and 3Zn(OH)_2_·2ZnCO_3_ anodes are excessively polarized during the charge process, resulting in low capacity. Figure 1e statistically shows the average discharge voltage and the voltage difference between the average charge and discharge values (referred to as polarization voltage) of these Zn^2+^-compound anodes. The average discharge voltage and polarization voltage exhibit similar rules: ZnF_2_ < ZnC_2_O_4_·2H_2_O < Zn_3_P_2_O_8_ < Zn(OH)_2_ < 3Zn(OH)_2_·2ZnCO_3_. However, the average discharge voltage improves slowly, while the polarization voltage increases rapidly. In order to understand the causes of the above phenomena, two representative anodes (ZnC_2_O_4_·2H_2_O and Zn(OH)_2_) were selected for theoretical analysis. The *pK_sp_* (*pK_sp_*=−log*K_sp_, K_sp_* being the solubility product) of ZnC_2_O_4_·2H_2_O is 8.86 which corresponds to dissolved *c*(Zn^2+^) of ∼3.7 × 10^−5^ mol L^−1^. While in Zn(OH)_2_ (*pK_sp_* = 16.5), only ∼1.9 × 10^−6^ mol L^−1^ Zn^2+^ could be dissolved, which is 20 times lower than that of ZnC_2_O_4_·2H_2_O. This also implies that the reduction of *c*(Zn^2+^) can indeed increase the battery voltage, but excessive reduction is not conducive to the kinetic process. It is worth noting that the large polarization voltage could also affect the output capacity of the cathode. Excessive polarization voltage tends to precipitate the rapid escalation in charge voltage. Consequently, upon reaching the cut-off voltage and terminating the charge process, the cathode capacity may not be fully released in time. Considering voltage and kinetic factors, the ZnC_2_O_4_·2H_2_O anode was finally selected for further mechanism research. The ZnC_2_O_4_·2H_2_O internal frame with large space consists of linear chain units, which are connected by hydrogen bonds (Fig. S7). Each linear chain is composed of planar oxalate ions and zinc ions as well as two water molecules perpendicular to the plane. This laid the foundation for the Zn/ZnC_2_O_4_·2H_2_O conversion reaction.

To further verify the universality of platform voltage increase, the ZnC_2_O_4_·2H_2_O anode was revealed in other battery systems. For example, ZnC_2_O_4_·2H_2_O anode combined with CH_3_COONa electrolyte and Na_3_V_2_(PO_4_)_3_ cathode can achieve a plateau voltage of ∼1.58 V (Fig. S8), while the common battery with the Zn anode only exhibits a plateau voltage of ∼1.43 V. Similarly, the operating plateau voltage of the ZnC_2_O_4_·2H_2_O-iodine battery is increased to ∼1.41 V from ∼1.26 V of the common Zn-iodine battery (Fig. S9). This not only further proves that the increase in plateau voltage is due to the introduction of the ZnC_2_O_4_·2H_2_O anode, but also shows that the ZnC_2_O_4_·2H_2_O anode is portable and not limited to a specific battery system.

### Structural and chemical evolution of ZnC_2_O_4_·2H_2_O anode

**Figure 2.**
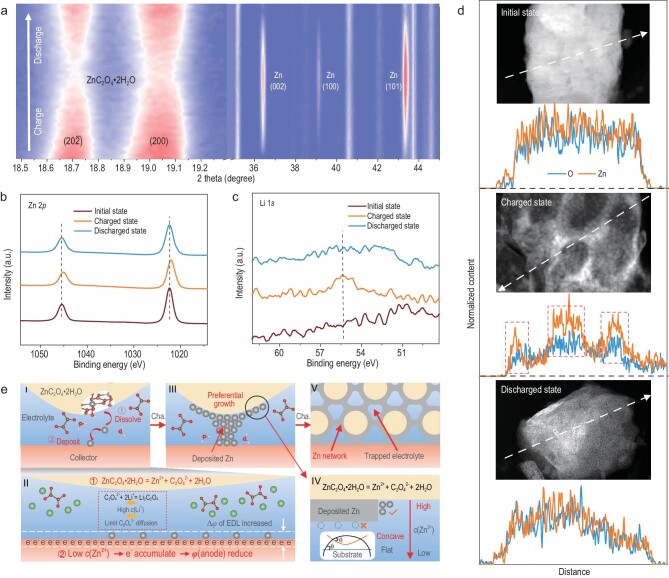
Analysis of the reaction mechanism of the ZnC_2_O_4_·2H_2_O anode. (a) *In-situ* XRD pattern of the ZnC_2_O_4_·2H_2_O anode. The *ex-situ* XPS spectra of (b) Zn 2*p* and (c) Li 1*s* in initial, charged, and discharged state. (d) The *ex-situ* scanning TEM and corresponding linear EDS results. (e) The specific process of the conversion reaction between Zn and ZnC_2_O_4_·2H_2_O. The specific process is divided into initial stage (inset **I**), growth stage (inset **III**) and final stage (inset **V**). The initial stage is subdivided into two steps of dissolution and deposition, and inset **II** is the detailed process analysis. Inset **IV** analyses the reason of preferential growth in inset **III**, mainly including the difference of *c*(Zn^2+^) and growth substrate.

*In-situ* X-ray diffraction (XRD) is employed to monitor the structural/chemical evolution of the ZnC_2_O_4_·2H_2_O anode (Fig. 2a). During the charging process, the ($20\bar{2}$) and (200) peaks of ZnC_2_O_4_·2H_2_O gradually weaken and the (002), (100) and (101) peaks of Zn gradually appear, which indicates the conversion reaction from ZnC_2_O_4_·2H_2_O to Zn. In the subsequent discharging process, the diffraction peaks of ZnC_2_O_4_·2H_2_O were re-enhanced and these Zn peaks disappeared, suggesting that this conversion reaction is highly reversible. Moreover, there is no additional phase generated (Fig. S10), demonstrating the simplicity of the anode reaction.

The changes of ionic valence during the reaction are also detected by *ex-situ* X-ray photoelectron spectroscopy (XPS). The Zn 2*p* (Fig. 2b) and O 1 *s* (Fig. S11) characteristic peaks shift to lower binding energy in the charged state, which indicates that Zn^2+^ in ZnC_2_O_4_·2H_2_O is reduced. After discharging, it reverts to its original state, which means a reversible conversion reaction between ZnC_2_O_4_·2H_2_O and Zn. The reversible changes observed in the C 1*s* peaks further confirm the reversible conversion reaction (Fig. S12). We observed a signal peak of Li 1*s* in the charging state (Fig. 2c), due to the combination of Li^+^ and generated C_2_O_4_^2−^ to form Li_2_C_2_O_4_ in the dry electrode. The presence and disappearance of the Li 1*s* peak suggests that the anode system maintains electric neutrality during the oxidation/reduction reaction between Zn^2+^ and Zn^0^. Because Li_2_C_2_O_4_ is soluble in water, there is no corresponding peak detected on the *in-situ* XRD patterns. In short, the reaction mechanism of the anode is the reversible conversion reaction between ZnC_2_O_4_·2H_2_O and Zn, as summarized in Equation 4.


(4) \begin{equation*}{\mathrm{Zn}}{{{\mathrm{C}}}_2}{{{\mathrm{O}}}_4} \cdot 2{{{\mathrm{H}}}_2}{\mathrm{O}} + 2{{{\mathrm{e}}}^ - } \rightleftharpoons {\mathrm{Zn}} + {{{\mathrm{C}}}_2}{\mathrm{O}}_4^{2 - } + 2{{{\mathrm{H}}}_2}{\mathrm{O}}\end{equation*}

The specific reaction process is further investigated via *in-situ* optical microscopy (OM), *ex-situ* cross-sectional scanning electron microscopy (SEM) and *ex-situ* transmission electron microscopy (TEM) studies. The snapshots of *in-situ* OM taken during the charge/discharge process are illustrated in Fig. S13. During the charging process, it is clearly visible that the light white ZnC_2_O_4_·2H_2_O on the surface of the substrate is gradually converted into deep black, which suggests the gradual growth of the electrodeposited Zn. The overall thickness of the anode remains unchanged, suggesting that the deposited Zn grows on the framework of the ZnC_2_O_4_·2H_2_O anode, which will be analyzed in detail later. From *ex-situ* cross-sectional SEM images (Fig. S14), it can be observed that numerous porous network-like structures are formed on the surface of ZnC_2_O_4_·2H_2_O in the charged state, which corresponds to the generation of Zn observed by *in-situ* OM. In the subsequent discharge process, the Zn with the network structure disappears and is converted back to ZnC_2_O_4_·2H_2_O particles. However, it is evident that the morphology of the newly-formed particles is significantly different from the original particles (Fig. S15). This change of morphology is due to the mechanism of conversion reaction, which explains the reasons for the variation of optical color in *in-situ* OM. The corresponding cross-sectional element distribution detected by energy dispersive spectroscopy (EDS) shows that the O element exhibiting the granular distribution at the charged stage is different from the distribution of Zn element, which suggests the growth of Zn metal. More microscopic morphological and structural evolution of ZnC_2_O_4_·2H_2_O particles during the charge and discharge process is further investigated by *ex-situ* scanning TEM (STEM). A distinct secondary phase of Zn metal can be detected at the charged state (Fig. S16). The corresponding linear EDS (STEM-LEDS) shows that the content of Zn elements in the network structure is significantly higher than that of bulk particles (Fig. 2d), which was proven by EDS mapping (Fig. S17a–c) results. And high-resolution TEM (HRTEM) images also detect the formation and disappearance of lattice fringes of Zn during cycling (Fig. S17d–f). This confirms the conversion reaction process between ZnC_2_O_4_·2H_2_O particle and Zn network.

Figure 2e analyzes and summarizes the specific process of the conversion reaction in detail. In the initial stage of conversion reaction, two processes occur: first, the dissolution equilibrium process of ZnC_2_O_4_·2H_2_O, and second, the deposition process of dissolved Zn^2+^ on the collector (inset **I** of Fig. 2e). In the first step, ZnC_2_O_4_·2H_2_O dissolves into Zn^2+^ and C_2_O_4_^2−^. Among them, Zn^2+^ migrate to the collector under the action of the electric field, while diffusion of C_2_O_4_^2−^ is bound by the high concentration of Li^+^ in the electrolyte (inset **II** of Fig. 2e). The main reason is that the solubility of Li_2_C_2_O_4_ is as low as ∼8 g/100 mL, and the corresponding solubility product constant (*K_sp_*) is ∼1.94 mol^3^ L^−3^. According to the solubility product formula of Li_2_C_2_O_4_ (Equation 5), the concentration of C_2_O_4_^2−^ in the electrolyte will be limited to a very low value of ∼0.05 mol L^−1^, because the *c*(Li^+^) in the electrolyte is ∼6 mol L^−1^.


(5) \begin{equation*}{{K}_{sp}}\!\left( {{\mathrm{L}}{{{\mathrm{i}}}_2}{{{\mathrm{C}}}_2}{{{\mathrm{O}}}_4}} \right) = c{{({\mathrm{L}}{{{\mathrm{i}}}^ + })}^2} \times c\left( {{{{\mathrm{C}}}_2}{\mathrm{O}}_4^{2 - }} \right)\end{equation*}

It means most of the dissolved C_2_O_4_^2−^ is confined near the ZnC_2_O_4_·2H_2_O particles, which is highly conducive to the conversion of Zn to ZnC_2_O_4_·2H_2_O during the subsequent discharge process, thus enabling the reversible conversion reaction of the anode. The coulombic efficiency (Fig. S18) and the Fourier transform infrared spectroscopy (FTIR) measurements (Fig. S19) further confirm the above conclusion. It should be noted from this process that not all Zn^2+^-compounds are suitable for low electrode potential anodes. Only Zn^2+^-compounds with a certain solubility in aqueous solution and whose dissolved anions remain stable in the solution and are restricted from shuttle can be used as anodes to reduce electrode potential.

In the second step, Zn^2+^ dissolved from ZnC_2_O_4_·2H_2_O are diffused to the surface of the collector to obtain electrons and then deposit into Zn. During this process, due to the low *c*(Zn^2+^), the rate of Zn^2+^ consuming electrons is far less than the rate that electrons migrate from the external circuit to the collector, leading to the accumulation of electrons at the anode. This accumulation leads to the decrease of the potential of the anode and the increase of the potential difference of the electrical double layer (EDL). As the conversion reaction goes on, the deposited Zn will grow along the surface of ZnC_2_O_4_·2H_2_O (inset **III** of Fig. 2e). The reasons for this characteristic growth can be summarized as follows (inset **IV** of Fig. 2e): first, the source of Zn^2+^ comes from the dissolution of ZnC_2_O_4_·2H_2_O particles. The *c*(Zn^2+^) on the surface of ZnC_2_O_4_·2H_2_O is the highest, so Zn^2+^ will preferentially deposit along the surface of ZnC_2_O_4_·2H_2_O. Second, the dissolved ZnC_2_O_4_·2H_2_O will construct a concave substrate for Zn^2+^ deposition. Compared to planar substrates, concave substrates have lower nucleation energy, which is more conducive to the growth of Zn. At the end of the conversion reaction, the surface of the ZnC_2_O_4_·2H_2_O is basically covered by a network-like Zn (inset **V** of Fig. 2e).

**Figure 3.**
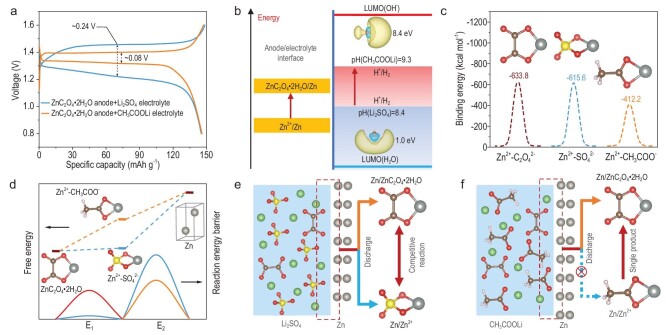
Investigation of electrolyte compatibility with the ZnC_2_O_4_·2H_2_O anode. (a) The comparison of GCD curve of a ZnC_2_O_4_·2H_2_O|LiFePO_4_ cell when matching Li_2_SO_4_ and CH_3_COOLi electrolyte. (b) Schematic diagram of energy level matching between the anode and electrolyte. (c) Comparison of binding energies between Zn^2+^ and C_2_O_4_^2−^, SO_4_^2−^, and CH_3_COO^−^. (d) The effect of binding energy differences on the charge process of the ZnC_2_O_4_·2H_2_O anode. Schematic diagram of the effect of (e) SO_4_^2−^ and (f) CH_3_COO^−^ on the discharge process of a ZnC_2_O_4_·2H_2_O anode.

Design of the anode alone is not sufficient for the normal operation of the above conversion reaction, because the compatibility between anode and electrolyte is crucial. Here we propose an important factor that can make this conversion reaction reversible, that is, the choice of CH_3_COOLi instead of Li_2_SO_4_ in electrolyte. From the specific reaction, there are two significant issues that will affect its reversibility and stability. First, due to the low *c*(Zn^2+^), the accumulation of electrons will be enabled, thus promoting the hydrogen evolution reaction. Second, compared to the concentration of anions in the electrolyte, the concentration of C_2_O_4_^2−^ dissolved during the charge process is very low. It is difficult for Zn^2+^ to combine effectively with C_2_O_4_^2−^ during the discharge process. To solve the above issues, the traditional SO_4_^2−^-based electrolyte is transformed into the CH_3_COO^−^-based electrolyte. As shown in Fig. 3a, compared to the Li_2_SO_4_ electrolyte, the CH_3_COOLi electrolyte owns lower charge voltage and higher discharge voltage by employing the LiFePO_4_ cathode and ZnC_2_O_4_·2H_2_O anode. Due to the fact that CH_3_COO^−^ are weak acid ions, its hydrolysis reaction can lead to an increase in the pH value of the electrolyte (pH(Li_2_SO_4_)=8.4, pH(CH_3_COOLi)=9.3, Fig. S20), further reducing the concentration of H^+^ and thus inhibiting the hydrogen evolution reaction (Fig. S21). The inhibition of water activity is also confirmed through molecular dynamics simulations (Fig. S22), and the percentage of hydrogen bonding in CH_3_COOLi electrolyte is lower than that in Li_2_SO_4_ electrolyte (Fig. S23). From the point of view of energy level, the elevated pH means an increase of the lowest unoccupied molecular orbital (LUMO) energy level of the electrolyte system (Fig. 3b), and this increase in the LUMO energy level is more compatible with the improvement of anode energy level caused by the decrease of electrode potential.

According to the calculation results of the binding energy between Zn^2+^ and different anions (Fig. 3c), the energy generated by the combination of Zn^2+^ and C_2_O_4_^2−^ (−633.8 kcal mol^−1^) is close to that of Zn^2+^ and SO_4_^2−^ (−615.6 kcal mol^−1^), but significantly higher than that of Zn^2+^ and CH_3_COO^−^ (−412.2 kcal mol^−1^). Considering that there are also no additional by-products generated in the Li_2_SO_4_ electrolyte (Fig. S24), it is speculated that the lower binding energy is a crucial reason why CH_3_COO^−^ is selected as anionic components of electrolyte. It can be analyzed in detail from the reaction process. During the charge process (Fig. 3d), ZnC_2_O_4_·2H_2_O first dissolves the solvated Zn^2+^, and then the solvated Zn^2+^ are reduced to Zn. Due to the high binding energy of SO_4_^2−^, the solvated zinc ions are difficult to reduce to Zn. For CH_3_COO^−^, low binding energy makes the solvated Zn^2+^ more easily reducible. Therefore, the charge voltage of CH_3_COOLi electrolyte is lower than that of Li_2_SO_4_ electrolyte. During the discharge process, Zn^2+^ stripped from the anode will preferentially bind to SO_4_^2−^ in Li_2_SO_4_ electrolyte due to the high concentration of SO_4_^2−^ (the binding energies of C_2_O_4_^2−^ and SO_4_^2−^ to Zn^2+^ are similar). Even if Zn^2+^ could bind to C_2_O_4_^2−^, the competitive reaction between SO_4_^2−^ and C_2_O_4_^2−^ will make Zn^2+^ bind again to SO_4_^2−^. Thus, the reversibility of the Zn/ZnC_2_O_4_·2H_2_O conversion reaction is disrupted (Fig. 3e). However, in CH_3_COOLi electrolyte, despite the high concentration of acetate ions, Zn^2+^ still preferentially binds to C_2_O_4_^2−^ due to the low bond energy of acetate ions, resulting in a single product of ZnC_2_O_4_·2H_2_O (Fig. 3f).

In short, the electrolyte compatible with the anode needs to meet two conditions. First, there is a requirement for energy level matching, where the LUMO energy level of the electrolyte needs to be higher than the chemical potential of the anode to prevent hydrogen evolution reactions. The second requirement is the binding energy matching between anions and zinc ions. The binding energy between the anions in the electrolyte and the zinc ions should be lower than the binding energy between the anions in the Zn^2+^-compounds and the zinc ions.

### Interfacial compatibility for high-voltage cathode

Although CH_3_COOLi electrolyte could be well compatible with the anode interface, it causes an oxygen evolution reaction and structural degradation of cathode materials under high operating voltages (inset **III** of Fig. 1a), thus resulting in a failure of battery (Fig. S25). To alleviate the contradiction between the cathode and anode interface, crowded micellar electrolyte with a water confinement effect has been proposed (inset **IV** of Fig. 1a). Raman spectroscopy (Fig. S26) indicates that the signal peak of free water is basically eliminated in crowded micellar electrolyte, which confirms that the crowded micellar electrolyte had a strong inhibition effect on free water. According to the molecular dynamics (MD) simulation (Fig. S27), a local electrostatic field is produced due to the negatively charged layer formed by oxygen ions on the surface of montmorillonite, which causes the polar water molecules nearby to arrange in order (bound water). With the increase of the distance from montmorillonite, the ordered water molecules will gradually transform into disordered water molecules (i.e. free water) because the effect of the electrostatic field is weaker than that of thermal motion. Inspired by the ‘molecular crowding’ strategy (Fig. S28) [32], the montmorillonite was used as a crowding agent to constrain the free water. The oxygen evolution reaction from the cathode side can be effectively inhibited (Fig. S29) because of the interface of cathode/crowded micellar electrolyte (Fig. S30). Although water activity is significantly inhibited, high ionic conductivity can still be maintained (Fig. S31).

**Figure 4.**
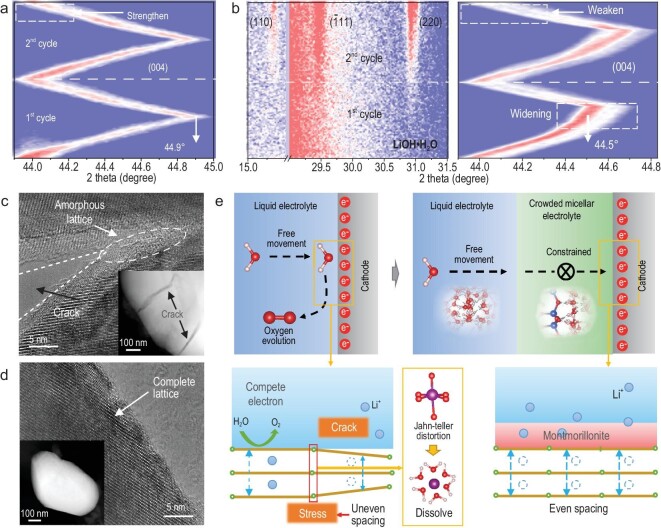
Exploration of the principle of crowded micellar electrolyte compatible with 2.0 V operating condition. *In situ* XRD spectral patterns of LiMn_2_O_4_ cathode under (a) crowded micellar electrolyte and (b) pure CH_3_COOLi electrolyte. The TEM and HRTEM characterizations of LiMn_2_O_4_ cathode after 3 cycles under the (c) pure CH_3_COOLi electrolyte and (d) crowded micellar electrolyte. (e) Corresponding principal analysis.

The crowded micellar electrolyte is the key factor for enabling the cathode to cycle reversibly with an operating platform voltage >2.0 V. The structure evolution of LiMn_2_O_4_ cathode with or without crowded micellar electrolyte is monitored by *in-situ* XRD. According to the changes of the (004) crystal plane (Fig. 4a), in the crowded micellar electrolyte, the peak position uniformly varies between 44.0° and 44.9° with no significant change in the width of the diffraction peak, which means that Li^+^ is uniformly and reversibly inserted or removed. The strongest peak appears at 44.0°, 44.5°, and 44.9°, suggesting the typical reaction mechanism of LiMn_2_O_4_ cathode (LiMn_2_O_4_→Li_0.5_Mn_2_O_4_→Mn_2_O_4_). There is no new phase in the reaction process (Figs S32 and S33), which manifests the stability and cycle reversibility of the cathode. However, in pure liquid electrolyte, the diffraction peak of (004) can only be shifted from 44.0° to 44.5°, and the width of the peak increases significantly when it is shifted to 44.5° (Fig. 4b). The cause of this phenomenon is mainly due to the oxygen evolution reaction. Under high voltage conditions, the oxygen evolution reaction and Li^+^ extraction reaction (Li_0.5_Mn_2_O_4_→Mn_2_O_4_) are a pair of competitive reactions. Li^+^ cannot continue to extract from the cathode, so the corresponding XRD patterns for the (004) peak can only shift to 44.5°. Moreover, due to the competition for electrons in the oxygen evolution reaction, the cathode is prone to uneven removal of Li^+^, which leads to differences in crystal plane spacing, as evidenced by the broadening diffraction peak. In addition, in pure liquid electrolyte, the intensity of the diffraction peak at the discharge stage gradually weakens, which suggests that the cathode material gradually dissolves [36]. The dissolution of the cathode is also accompanied by the production of LiOH·H_2_O by-products (Fig. S34). It is well known that such by-products are very common in cathode materials of lithium-ion batteries, which is generated by the reaction between lithium produced by the de-lithiation process of the cathode material and active water [37]. The high activity of water and the dissolution of active substances are beneficial for the formation of these by-products [38].

The TEM and HRTEM characterizations of morphological evolution of LiMn_2_O_4_ cathode after 3 cycles are completely consistent with the above analysis. Obviously, in liquid electrolyte, numerous microcracks (Fig. S35) and by-products (Fig. S36) can be observed. And the HRTEM image shows that the crystal lattice has become amorphous near the crack (Fig. 4c). However, the morphology and lattice stripes of LiMn_2_O_4_ particles (Fig. 4d) can remain in the crowded micellar electrolyte, which is pretty much in its original state (Fig. S37). Figure 4e vividly illustrates the reaction evolution process. In liquid electrolyte, due to the competition of oxygen evolution reactions, uneven Li^+^ is removed from the LiMn_2_O_4_ crystal structure, resulting in the internal stress and the Jahn–Teller distortion of the [MnO_6_] polyhedron [39,40]. However, in the crowded micellar electrolyte, the stability of the cathode has been greatly improved due to the confinement of activated water and the suppression of the oxygen evolution reaction. The corresponding schematic diagram provides a detailed explanation of the function of the crowded micellar electrolyte, which greatly eliminates side reactions on the cathode surface.

According to the design above, a battery scheme of ZnC_2_O_4_·2H_2_O (anode) | CH_3_COOLi (anodic electrolyte) | montmorillonite (cathodic electrolyte) | LiMn_2_O_4_ (cathode) was designed, as shown in Fig. S38. Compared with the conventional Zn (anode) | Li_2_SO_4_/ZnSO_4_ (electrolyte) | LiMn_2_O_4_ (cathode) system, this novel battery achieves an operating platform voltage >2.0 V, a coulombic efficiency near 100%, a 17.7% increase of energy density based on cathode material mass (Fig. S39), as well as better self-discharge performance (Fig. S40). However, it will suffer from severe oxygen evolution and ultra-low coulomb efficiency when it lacks the crowded micellar electrolyte, although the platform voltage can reach >2.0 V. Figure S41 shows the voltage profiles of our designed battery system, which exhibits high reversibility. This novel battery could maintain more than 100 cycles with a capacity retention of 95% (Fig. S42), indicating the promising prospects of our high-voltage AZIBs.

### Technological exploration of practical Ah-scale pouch cell

It is well known that the realistic pouch cell under large area capacity and high loading is necessary in practical application [41,42]. Nevertheless, there are great scientific and technical barriers from the coin cell design to the final attempt to make a breakthrough in Ah-scale technology [15]. These critical and self-contradictory issues were thoroughly explored in this work, which is believed to provide a novel way for follow-up research work. To investigate the failure mechanisms of the pouch cell, ten possible factors affecting the performance of the pouch cell were analyzed, including anodic collector area, zinc powder content in the anode, hydrophilicity of anodic binder, acetylene black content in the anode, Li^+^-compound content in the anode, crack resistance of cathodic electrolytes, hydrophilicity of cathodic binder, Li^+^-compound content in the cathode, cathodic collector area, cathodic collector oxidizability. As shown in Fig. S43a, the assembly method employed in the coin cell is completely unsuitable for the pouch cell. Moreover, modulating a single factor, such as adjusting the type of collector, the composition of the anode or cathode, or the preparation method of the cathodic electrolyte, is insufficient to achieve the desired performance of the pouch cell (Fig. S43b–l), which is intricately influenced by a combination of multiple factors. However, in the actual experimental process, it is very difficult to determine critical factors through limited experiments.

**Figure 5.**
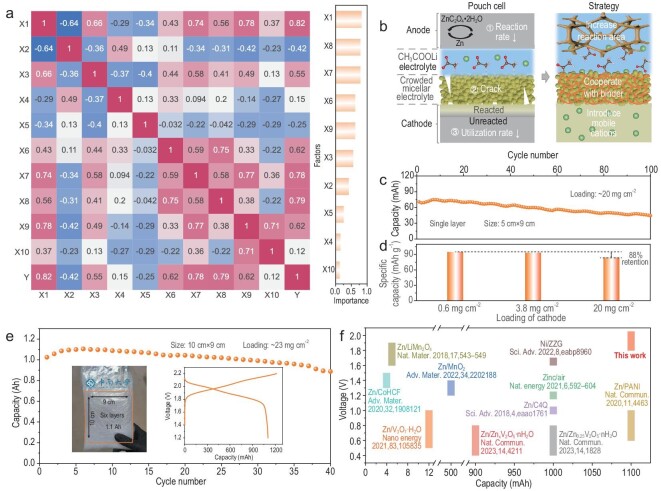
Exploration of the manufacturing technology of a pouch cell. (a) The heatmap of Pearson correlation coefficient between two variables of ten factors (X1: anodic collector area; X2: zinc powder content in the anode; X3: hydrophilicity of anodic binder; X4: acetylene black content in the anode; X5: Li^+^-compound content in the anode; X6: crack resistance of cathodic electrolyte; X7: hydrophilicity of cathodic binder; X8: Li^+^-compound content in the cathode; X9: cathodic collector area; X10: cathodic collector oxidizability) and the importance ranking of the effect of factors on performance. (b) The issues in the production process of pouch cells and the proposed strategy in this work. (c) The cycle performance of a single layer pouch cell with a size of 5 cm × 9 cm at 1.11 mA cm^−2^. (d) The relationship between the specific capacity and loading. (e) The cycle performance of a six-layer pouch cell with a size of 10 cm × 9 cm at 0.44 mA cm^−2^ (inset: the pouch cell device with the capacity of 1.1 Ah and the GCD curve). (f) Performance comparison of the pouch cells.

To identify the main influencing factors on the performance of the pouch cell, relevant data pre-processing methods of machine learning (ML) have been adopted [43,44]. By calculating the Pearson correlation coefficient between these variables (Fig. 5a), several main factors affecting the performance of the pouch cell such as anodic collector area, crack resistance of cathodic electrolyte, hydrophilicity of cathodic binder, and Li^+^-compound content of the cathode, were revealed. Figure 5b summarizes the failure mechanisms and optimization strategies of the pouch cell. Sluggish reaction rate and large polarization, especially under high loading, are the main challenges for ZnC_2_O_4_·2H_2_O/Zn conversion reaction, which can be resolved by increasing the reaction area (e.g. three-dimensional anodic collector). The uneven expansion of crowded micellar electrolyte in the process of water absorption will make it easy to crack, leading to the loss of water confinement. By introducing long-chain organic molecules with good water dispersibility, such as polytetrafluoroethylene (PTFE), to connect dispersed montmorillonite particles, would increase the structural stability of crowded micellar electrolyte. High loading of active material will suffer from low utilization, particularly, the solid-solid interface impedes the penetration of working ions into the cathode framework, which is averse to exciting the capacity of the pouch cell. Increasing ion conductivity by mixing additional hydrophilic Li^+^-compound such as lithium polyacrylate (PAA-Li) binder can effectively address this challenge.

Based on the above technological exploration, the pouch cell with cathode loading >20 mg cm^−2^ and large capacity of 1.1 Ah has been successfully fabricated. A single layer pouch cell with the size of 5 cm × 9 cm exhibits ∼80 mAh with a 2.0 V discharge platform (Fig. S44), 100 stable cycles (Fig. 5c) and good rate performance (Fig. S45). Importantly, there is an 88% capacity retention under high loading of more than 20 mg cm^−2^ compared to that of 0.6 mg cm^−2^ (Fig. 5d). The performance under high cathode loading also indirectly indicates that the conversion-type anode could maintain stable reaction across different areal capacity. Moreover, the above pouch technology is also applicable to the larger size pouch cell and multilayered stacking configurations. The Ah-level pouch cell with six-layer stacking configuration (10 cm × 9 cm) was further explored, which exhibits 1.1 Ah with an 86% capacity retention after 40 cycles at 0.44 mA cm^−2^ (Fig. 5e). Although the loading reaches Ah scale, the 2.0 V discharge platform remains (Fig. 5e, inset), further proving the superiority of the pouch technology in this work.

Compared to the previous reported performance of pouch cells with Zn anodes, such as Zn/Zn_0.25_V_2_O_5_·nH_2_O (0.4–0.8 V, 1000 mAh) [45], Zn/Zn_x_V_2_O_5_·nH_2_O (0.4–0.8 V, 900 mAh) [46], Zn/MnO_2_ (1.2–1.4 V, 500 mAh) [47], Zn/air (1.15–1.25 V, 1000 mAh) [48], Zn/C4Q (0.95–1.05 V, 1000 mAh) [49], Zn/PANI (0.6–1.0 V, 1100 mAh) [50], Zn/CoHCF (1.3–1.5 V, 4 mAh) [51], Ni/ZZG (1.6–1.7 V, 1000 mAh) [22], Zn/LiMn_2_O_4_ (1.6–1.9 V, 5 mAh) [52], and Zn/V_3_O_7_·H_2_O (0.5–1.0 V, 12 mAh) [53], the high discharge platform voltage (1.8–2.05 V) and high capacity (1.1 Ah) could be obtained simultaneously for our system (Fig. 5f), which would provide valuable guidance for the design of high energy density devices.

## CONCLUSIONS

In this work, an innovative conversion-type ZnC_2_O_4_·2H_2_O anode guided by the electrode potential was designed, towards high-voltage near-neutral AZIBs with an operating platform voltage beyond 2.0 V. A meticulous consideration of the modified Nernst relation establishes a real relationship between ion concentration of Zn^2+^ and the anode electrode potential. A reversible transformation between ZnC_2_O_4_·2H_2_O particles and three-dimensional Zn metal networks, which was well-matched by CH_3_COO^−^-based electrolyte, was revealed via *in-situ* XRD, scanning TEM and linear EDS, etc. By combining with montmorillonite-based crowded micellar electrolyte, the cracks in cathode electrode and oxygen evolution reaction in the cathodic interface under high-voltage operation have been prevented. The button cell obtained a capacity retention of 95% after 100 cycles. The practical pouch cell achieved an 88% capacity retention under high loading of >20 mg cm^−2^ and stabilize lifespan for 100 cycles. Furthermore, a 1.1 Ah pouch cell was further explored, which exhibited an 86% capacity retention after 40 cycles at 0.44 mA cm^−2^. This work provides new insights into the design of high-voltage near-neutral AZIBs from the perspective of novel conversion-type and electrolyte regulation.

## METHODS

Detailed materials and methods are available in the online Supplementary data.

## Supplementary Material

nwae181_Supplemental_Files
