# Supplementary material for: Conversion-type anode chemistry with interfacial compatibility toward Ah-level near-neutral high-voltage zinc ion batteries
Source: Natl Sci Rev. 2024 May 25;11(7):nwae181. doi: 10.1093/nsr/nwae181 (PMC11193386; doi:10.1093/nsr/nwae181)
Supplement: nwae181_Supplemental_Files [file nwae181_supplemental_files.zip › Supplementary data.pdf]

Supporting information for

Conversion-type anode chemistry with interfacial compatibility toward Ah-level near-neutral high-voltage zinc ion batteries

Shan Guo [1], Liping Qin[2], Jia Wu[1], Zhexuan Liu[1], Yuhao Huang[1], Yiman Xie[3] Guozhao Fang [1]*, Shuquan Liang [1]

[1] School of Materials Science and Engineering, Key Laboratory of Electronic Packaging and Advanced Functional Materials of Hunan Province, Central South University, Changsha 410083, China. E-mail addresses: fg_zhao@csu.edu.cn.

[2] College of Biological and Chemical Engineering, Guangxi University of Science and Technology, Guangxi 545006, China

[3] Information and Network Center, Central South University, Changsha 410083, China

Experimental procedures

Materials

The CH_3COOLi electrolyte is prepared by mixing the CH_3COOLi powder with the deionized water at the mass/volume ratio of 2g : 5mL. For the coin cell, the crowded micellar electrolyte is fabricated by the method of compression molding: 0.2 g mixture of montmorillonite and guar gum is pressed into round piece (diameter = 15mm). The purpose of adding guar gum is to increase the toughness of the round piece and prevent crowded micellar electrolyte failure caused by cracking. For the pouch cell, the crowded micellar electrolyte is manufactured by the means of rolling fabrication: Firstly, montmorillonite, CH_3COOLi and polytetrafluoroethylene (PTFE) preparation are mixed according to mass ratio (e.g. 7:2:1 or 5:5:1). The function of CH_3COOLi component is to increase the ionic conductivity of the crowded micellar electrolyte. The PTFE acts as the adhesive to improve the toughness. Then, the slurry mixture is fabricated to the large-area crowded micellar electrolyte by the simple rolling method.

Electrochemical measurements

For the coin cell, the preparation process of the cathode film is as follows: $LiMn_2O_4$, acetylene black, and polyvinylidene difluoride (PVDF) at the mass ratio of 7:2:1 is mixed evenly in N-methylpyrrolidone (NMP) solvent. The mixed slurry is coated on stainless steel foil and dried in 80 ℃ vacuum oven. The preparation of anode film is to mix $ZnC_2O_4 \cdot 2H_2O$ and PVDF at the mass ratio of 9:1 in NMP solvent. After mixing evenly, the slurry is coated on zinc foil, and then it is dried in 80 ℃ vacuum oven. The coin cell is assembled by using 2016 R coin-type cells in the order of cathode film, crowded micellar electrolyte, CH_3COOLi electrolyte, and anode film.

For the pouch cell, the cathode film is prepared by dispersing $LiMn_2O_4$, acetylene black and lithium polyacrylate (PAA-Li) at the mass ratio of 7:2:1 in water solvent and then coating the slurry on carbon cloth. To fabricate the anode, $ZnC_2O_4 \cdot 2H_2O$, acetylene black and PVDF are mixed in NMP solvent at the mass ratio of 7:2:1. The mixed slurry is then coated on the zinc foam. Finally, the coated cathode or anode films are dried in the 80 ℃ vacuum oven. The single layer pouch cell is assembled using a similar method to coin cell, and then packaged in a vacuum-packed bag. Multilayer pouch cell is assembled using stacking technology. Six cathodes, six anodes, six crowded micellar electrolytes, nine separators injected by CH_3COOLi electrolyte are stacked layer by layer.

Simulations

The molecular dynamics simulations are performed by the LAMMPS software. For the model of Li_2SO_4 electrolyte, 555 water molecules, 40 Li^+, and 20 SO_4^{2-} are added. In the model of CH_3COOLi electrolyte, 555 water molecules, 60 Li^+, and 60 CH_3COO^- are added. When simulating micelle structure of montmorillonite, 2400 water molecules and 1 montmorillonite particle are added. For the simulation of crowded micellar electrolyte, 2 montmorillonite particles and 484 water molecules are added. A simulation of 2ns by using 1 fs time step in the isothermal-isobaric ensemble (NPT) ensemble is firstly conducted at 1 atm pressure and 300 K. Then, these models are equilibrated under the canonical ensemble (NVT) condition at 300 K by using 0.1 fs time step for 20 ps.

Figures and captions

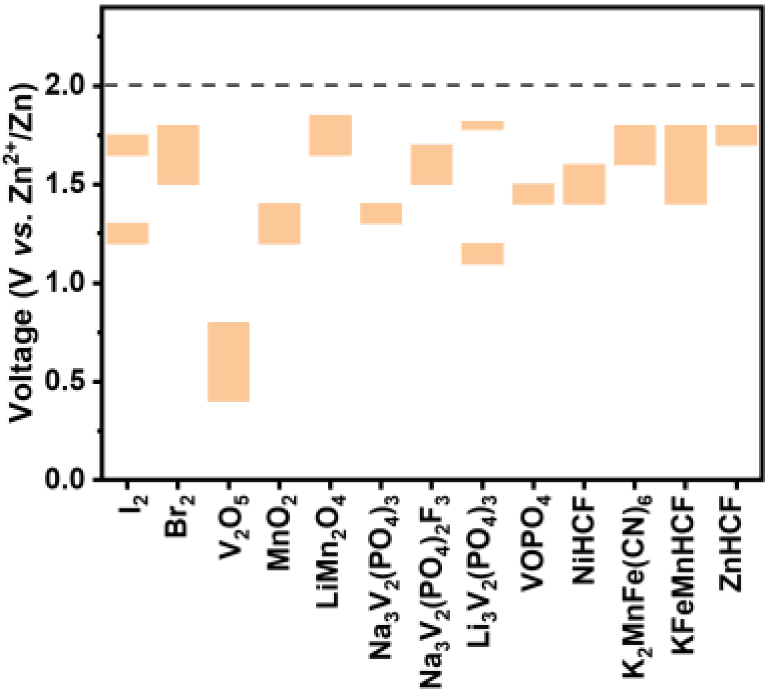

Figure S1. The platform voltages of most reported near-neutral ZIBs.

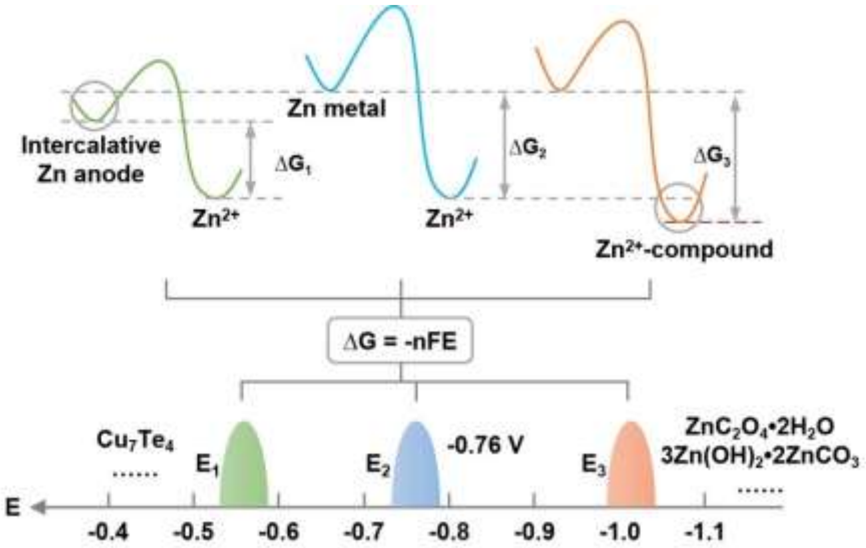

Figure S2. The influence of Gibbs free energy change on the anode electrode potential.

Gibbs free energy change (ΔG) of the anodic electrode potential could be expressed as **Eq. S1**:

$$\Delta G = -nFE \tag{S1}$$

ΔG represents the Gibbs free energy change, n represents the number of electrons transferred in the electrode reaction, F represents the Faraday constant, and E represents the electrode potential of the anode. Considering that ΔG is actually the Gibbs free energy of the reduction state minus the Gibbs free energy of the oxidation state, the electrode potential of the anode can be adjusted by regulating the Gibbs free energy of the reduction state or the oxidation state. For the intercalative Zn anode, the free energy of the intercalative Zn anode at reduction state is lower than that of the zinc metal, so the electrode potential of the intercalative Zn anode (for example, -0.56 V of Cu_7Te_4 anode[2]) is higher than that of the standard zinc metal anode (-0.76 V). For the Zn^{2+}-compound anode, the anions with stronger binding energy (such as $C_2O_4^{2-}$ or CO_3^{2-}) will bind to the free state Zn^{2+} to produce the Zn^{2+}-compound precipitation (ZnC_2O_4•$2H_2O$, $3Zn(OH)_2$•$2ZnCO_3$, etc) at oxidation state. The generation of stronger binding energy implies a further decrease in the Gibbs free energy compared to free state Zn^{2+}, leading to higher ΔG. Therefore, the electrode potential of the Zn^{2+}-compound anode (~ -1.0V) is generally lower than that of the standard zinc metal anode.

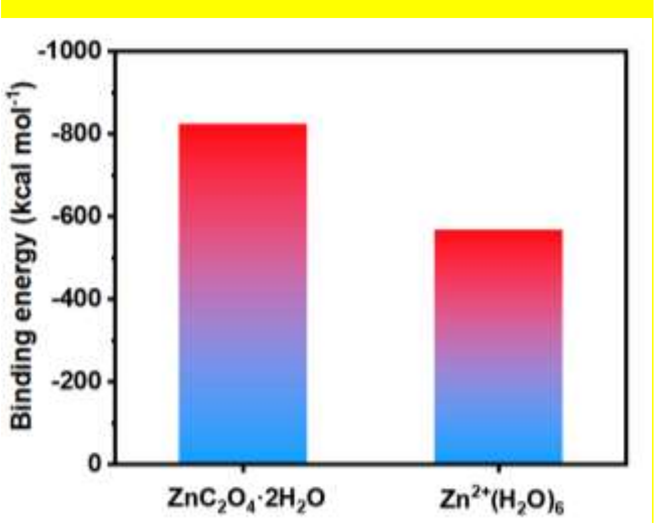

Figure S3. The binding energies of ZnC_2O_4 • $2H_2O$ and the typical solvation structure of free state Zn^{2+}.

The calculation results indicate that the binding energy of ZnC_2O_4 • $2H_2O$ is greater than the typical solvation binding energy of free zinc ions. This implies that the formation of Zn^{2+}-compound would result in the larger Gibbs free energy change (ΔG) than the free zinc ions.

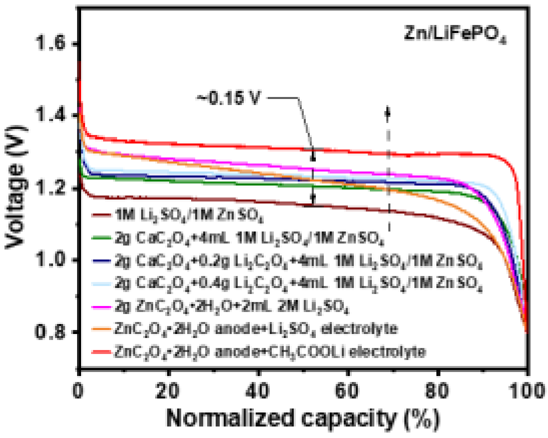

Figure S4. The influence of anode and electrolyte regulation on the discharge curve of $LiFePO_4$. b, Comparison of discharge curves of $Na_3V_2(PO_4)_3$ by matching Zn anode and ZnC_2O_4 • $2H_2O$ anode.c, Comparison of discharge curves of I_2 by matching Zn anode and ZnC_2O_4 • $2H_2O$ anode.

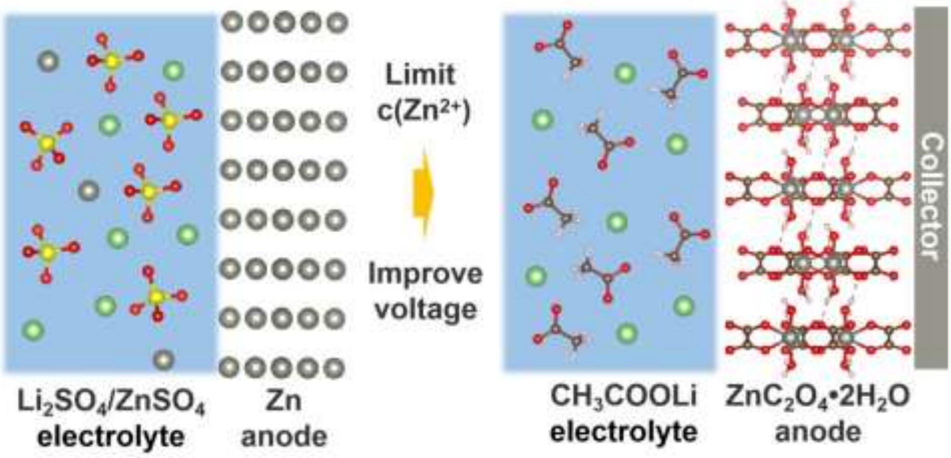

Figure S5. Schematic illustration of regulation mechanism of anode voltage.

After replacing the Zn anode with ZnC_2O_4•$2H_2O$ anode, the corresponding electrolyte needs to be replaced from SO_4^{2-}-based electrolyte to CH_3COO^--based electrolyte. Its main purpose is to prevent the influence of SO_4^{2-} on ZnC_2O_4•$2H_2O$ anodes. Due to the high binding energy between SO_4^{2-} and Zn^{2+}, they will participate in the competitive reaction between $C_2O_4^{2-}$ and Zn^{2+}, affecting the reversibility of the anodic reaction. The binding energy between CH_3COO^- and Zn^{2+} is weak, which does not hinder the normal operation of the anodic reaction.

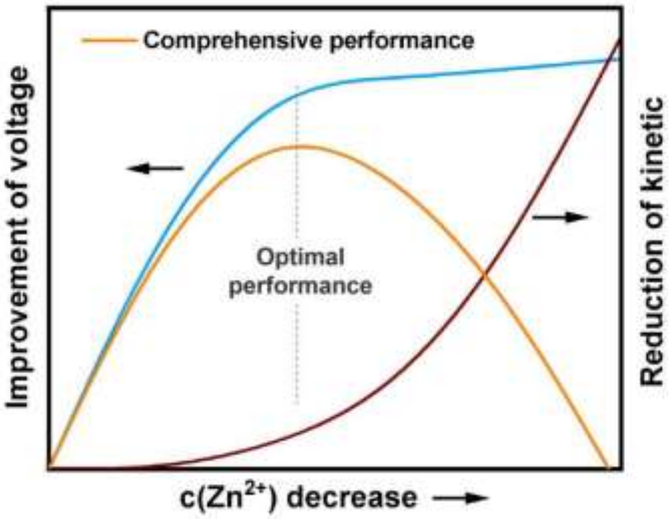

Figure S6. The effect of the reduction of $c(Zn^{2+})$ on comprehensive electrochemical performance.

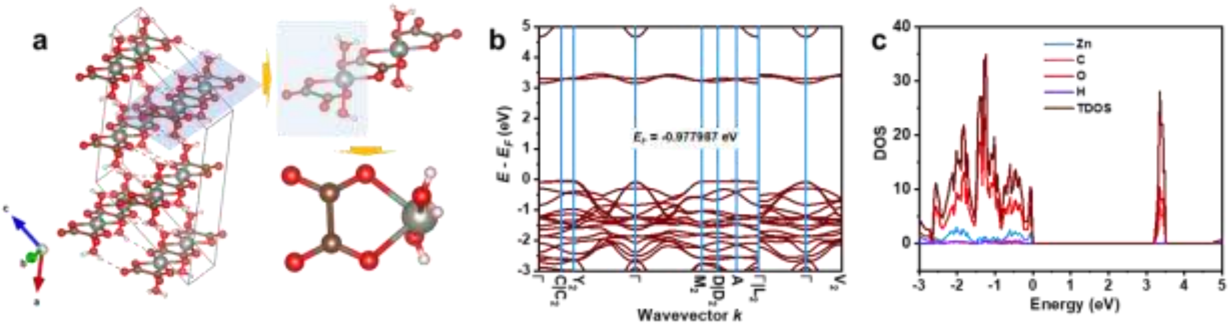

Figure S7. The crystal and electronic structure of ZnC_2O_4•$2H_2O$. **a**, The crystal structure. **b**, The band structure. **c**, The density of states.

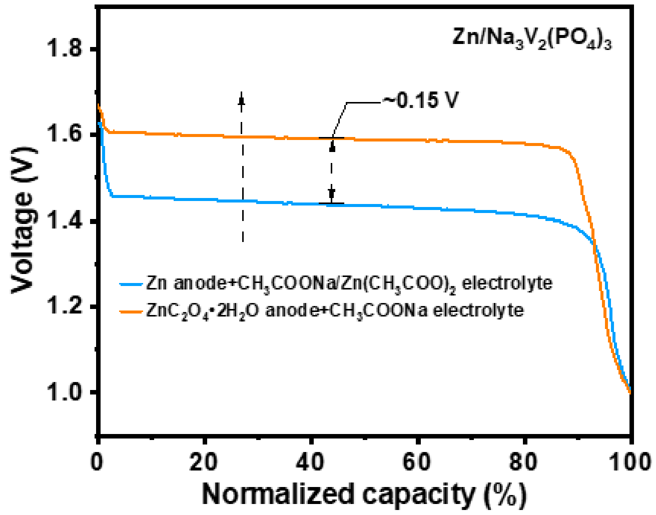

Figure S8. Comparison of discharge curves of $Na_3V_2(PO_4)_3$ by matching Zn anode and ZnC_2O_4 • $2H_2O$ anode.

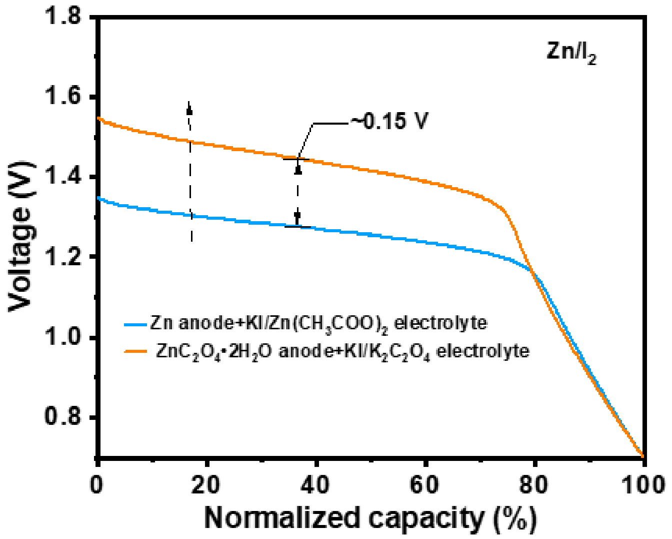

Figure S9. Comparison of discharge curves of I_2 by matching Zn anode and $ZnC_2O_4 \cdot 2H_2O$ anode.

The variation in electrolyte formula between zinc-iodine batteries and other battery systems is attributed to differences in their energy storage mechanisms. For aqueous lithium-ion or aqueous sodium-ion batteries, the cathode reaction only involves a simple insertion/extraction process of cations in the electrolyte, thus the anion of electrolyte only needs to contain CH_3COO^-. However, for zinc-iodine batteries, the cathodic reaction primarily involves the conversion of iodide ions. Therefore, the anions in the electrolyte not only contain CH_3COO^-, but also I^-. According to the principle of compatibility between electrolyte and anode, the presence of additional I^- in the electrolyte would disrupt the reversible conversion reaction at the anode. To enhance the reversibility of the anode, additional $C_2O_4^{2-}$ are introduced into the electrolyte to increase their competitiveness. Therefore, the electrolyte formula of zinc-iodine batteries differs from others.

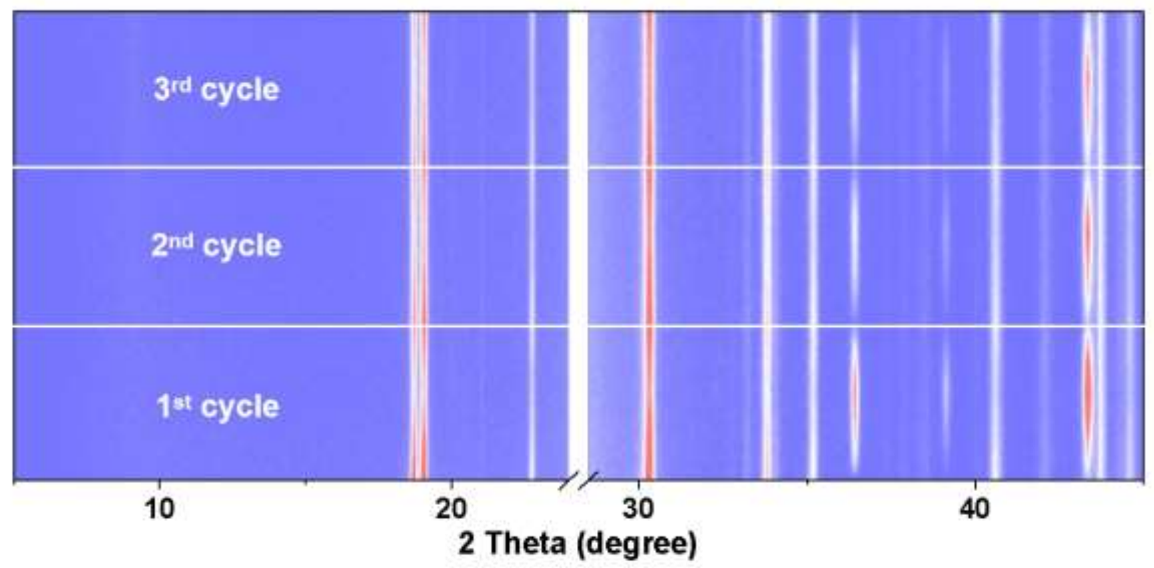

Figure S10. The *in-situ* XRD spectra of ZnC_2O_4•$2H_2O$ anode in the first 3 cycles.

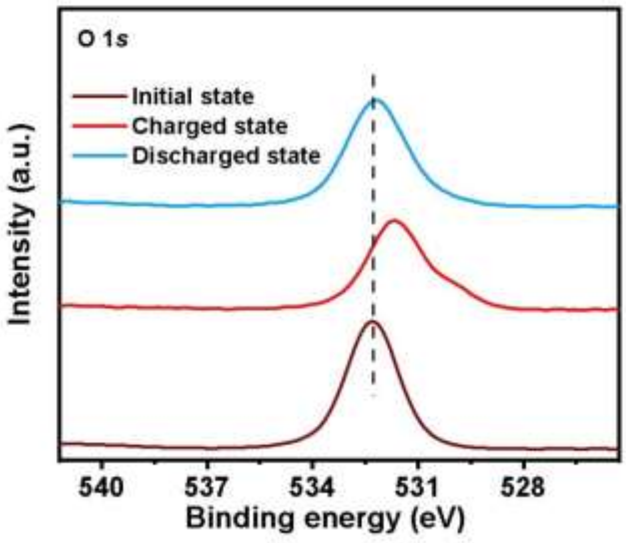

Figure S11. The *ex-situ* XPS spectra of O 1s in initial, charged and discharge state.

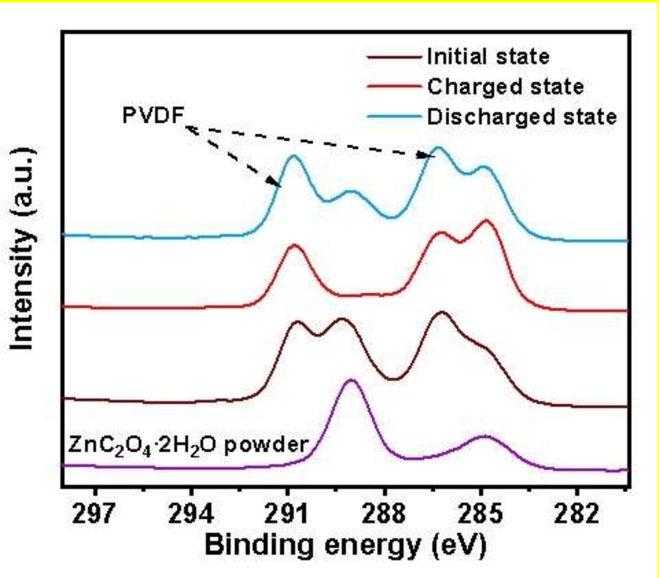

Figure S12. The *ex-situ* XPS spectra of C 1s.

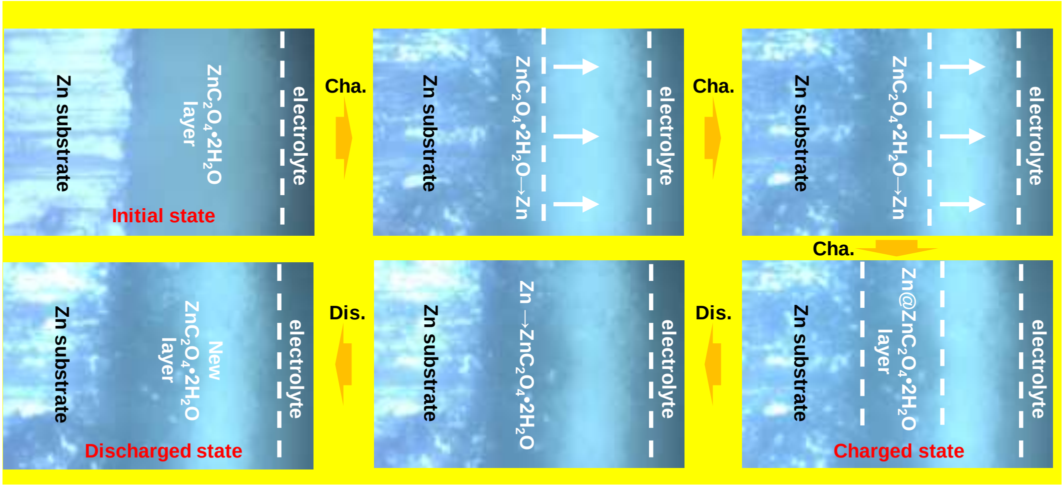

Figure S13. The snapshots of *in-situ* OM of $ZnC_2O_4 \cdot 2H_2O$ anode during the initial change/discharge process.

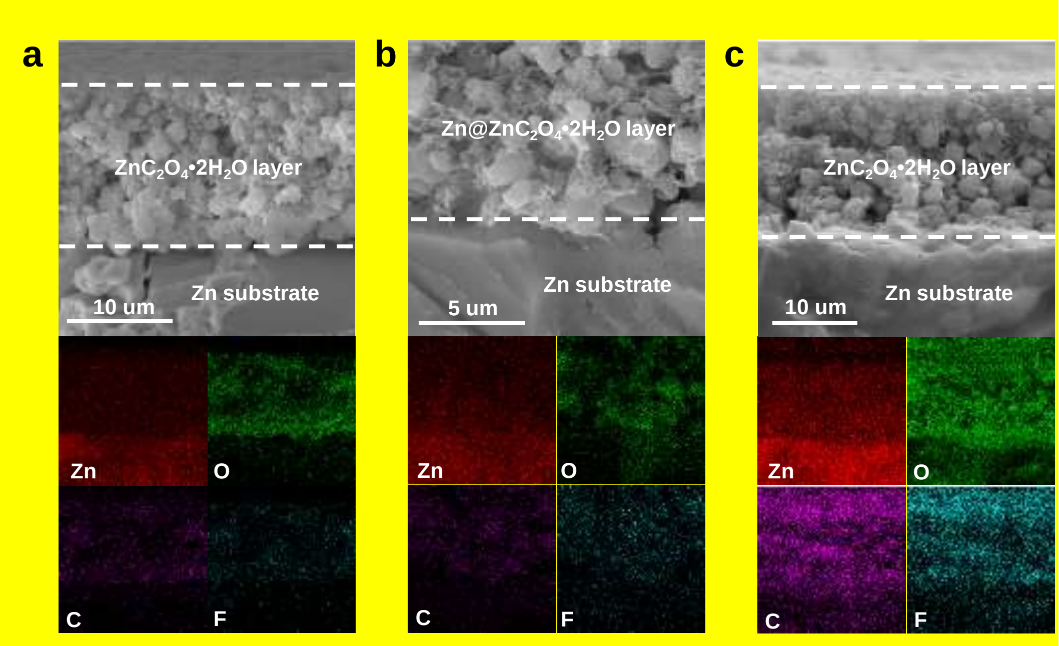

Figure S14. ***Ex-situ*** **cross-sectional SEM and corresponding EDS image of $ZnC_2O_4 \cdot 2H_2O$ anode during the first change/discharge process** (**a,** In the initial state. **b,** In the charged state. **c,** In the discharged state).

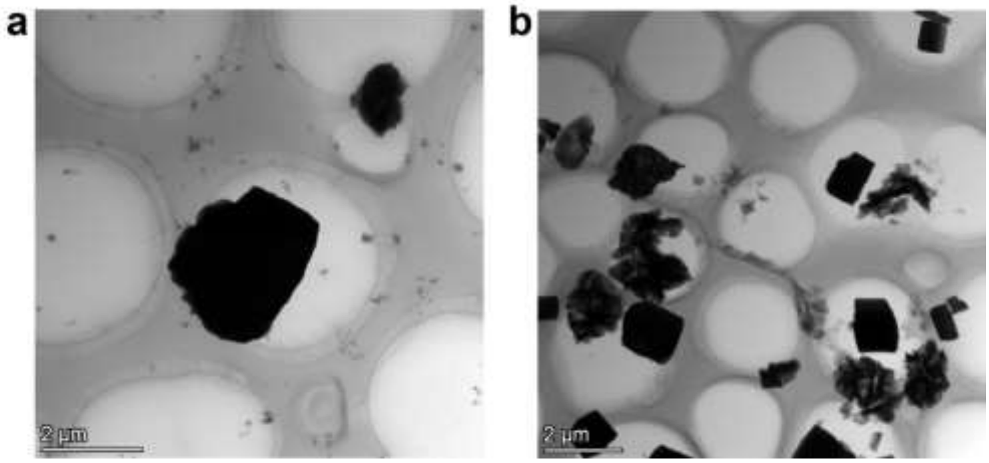

Figure S15. Comparison between the initial morphology of the anode particles and their morphology after cycling. (a) The initial morphology, (b) The morphology after cycling.

It can be observed that the morphology after cycling exhibits the more regular shape. The reason for the change in morphology is due to the mechanism of the anodic reaction, which is governed by the conversion reaction. During the conversion reaction process, $ZnC_2O_4 \cdot 2H_2O$ particles undergo dissolution during the charge process and re-growth during the discharge process. The re-grown $ZnC_2O_4 \cdot 2H_2O$ tends to adopt the more regular morphology due to thermodynamic stability principles.

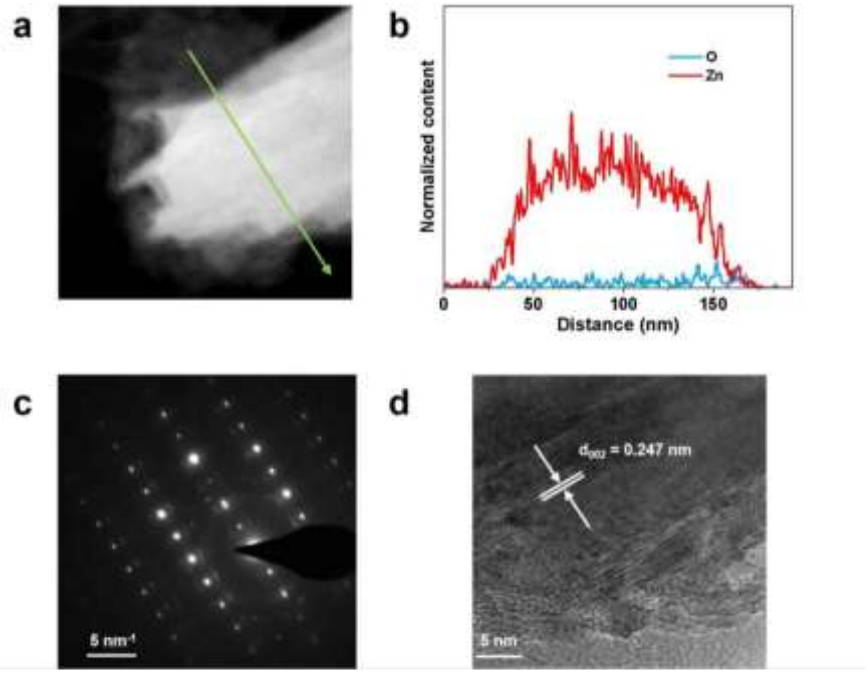

Figure S16. The characterization of Zn particle in charged state. a, The STEM image. **b,** Corresponding linear EDS results. **c,** Selected area electron diffraction (SAED) pattern. **d,** HRTEM image.

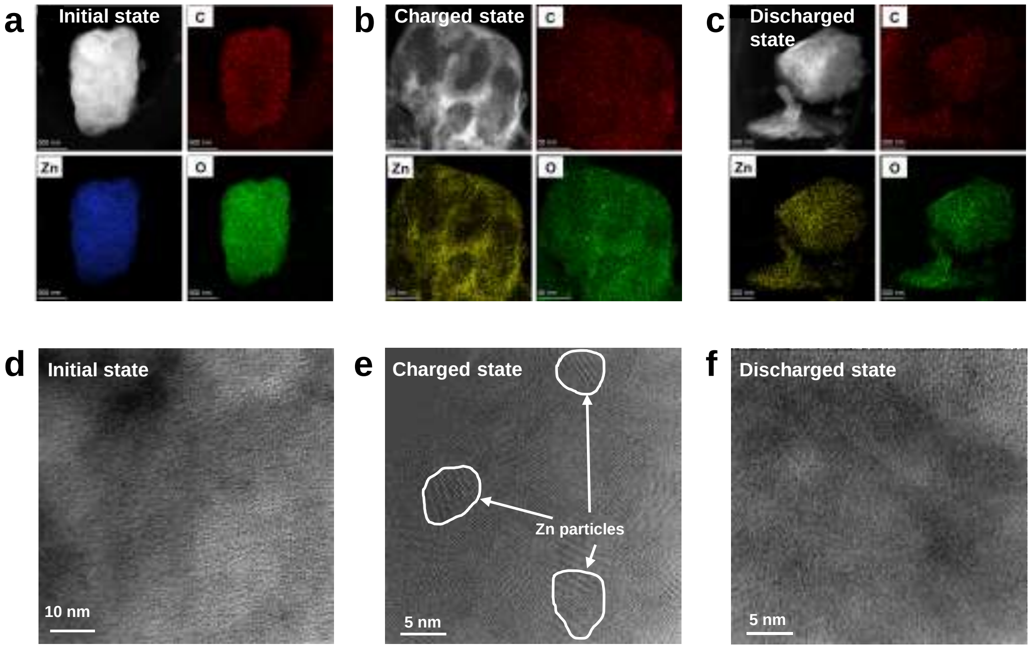

Figure S17. The *ex-situ* EDS mapping and HRTEM of ZnC_2O_4 2H_2O anode. The EDS mapping of ZnC_2O_4 2H_2O particle during different states (**a,** In the initial state. **b,** In the charged state. **c,** In the discharged state). *Ex situ* HRTEM images during different states (**d,** In the initial state. **e,** In the charged state. **f,** In the discharged state).

Based on the specific process of zinc deposition, it is evident that in order to render the anodic

reaction more reversible, $ZnC_2O_4 \cdot 2H_2O$ particles must not be entirely consumed during the charging process. Instead, a portion of their framework needs to be preserved to facilitate the growth of metallic zinc. Therefore, the area capacity generated by the deposited zinc needs to be smaller than the theoretical capacity of the $ZnC_2O_4 \cdot 2H_2O$ conversion reaction.

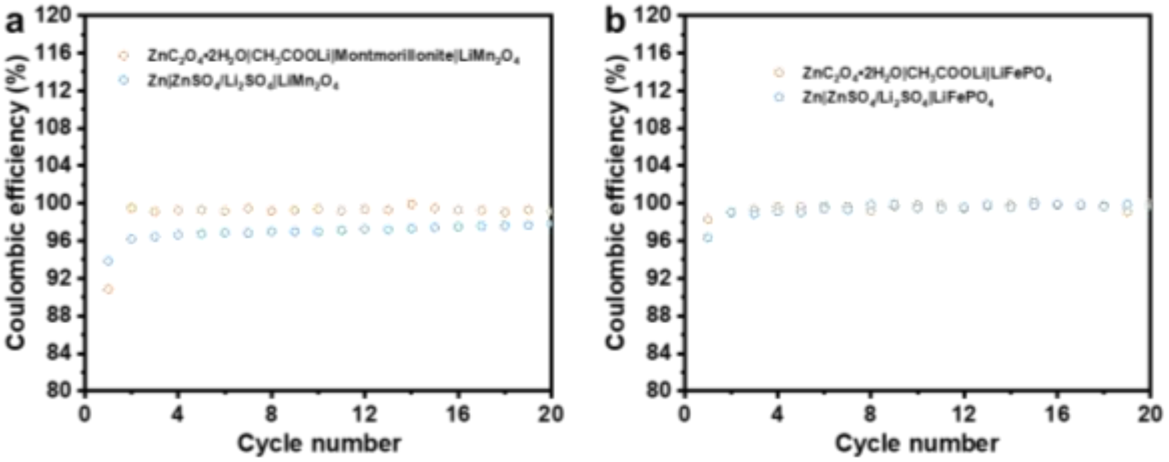

Figure S18. The comparison of coulombic efficiency. (a) The comparison of coulombic efficiency between the designed battery system and the conventional $Zn/LiMn_2O_4$ battery; (b) The comparison of coulombic efficiency between the $ZnC_2O_4 \cdot 2H_2O/LiFePO_4$ battery and $Zn/LiFePO_4$ battery.

The suppression of shuttle effects for $C_2O_4^{2-}$ can be evidenced by the coulombic efficiency of battery. **Figure S18** illustrates the coulombic efficiency of the battery system designed in this work and the $ZnC_2O_4 \cdot 2H_2O/LiFePO_4$ battery system at the first 20 cycles. Both systems employ $ZnC_2O_4 \cdot 2H_2O$ as the anode. By examining their coulombic efficiency, the shuttle behavior of $C_2O_4^{2-}$ can be indirectly assessed. **Figure S18a** depicts the comparison of coulombic efficiency between the designed battery system and the conventional $Zn/LiMn_2O_4$ system. The designed battery system demonstrates nearly 100% coulombic efficiency, which is significantly superior to the comparison sample. The near 100% Coulombic efficiency implies that the $C_2O_4^{2-}$ released during the charge process are fully recombined during the discharge process, suggesting the absence of $C_2O_4^{2-}$ shuttle phenomenon. The near 100% Coulombic efficiency observed in **Figure S18b** for the $ZnC_2O_4 \cdot 2H_2O/LiFePO_4$ system further confirms the aforementioned conclusion. In short, the overall coulombic efficiency results of the battery indicate the shuttle phenomenon of $C_2O_4^{2-}$ does not occur.

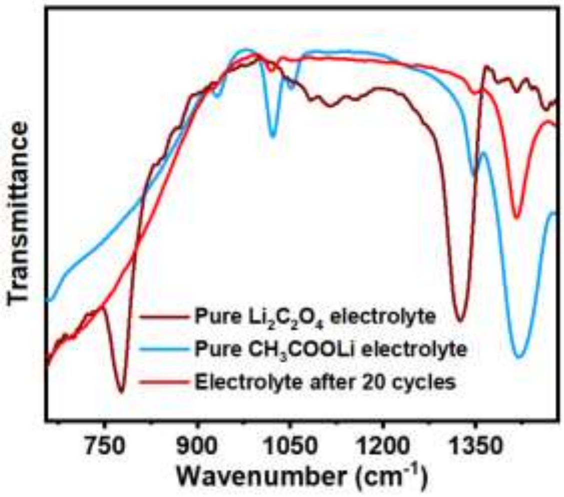

Figure S19. FTIR measurements on the electrolyte of the ZnC_2O_4 • $2H_2O/LiFePO_4$ battery after 20 cycles.

Apart from the analysis of coulombic efficiency, whether $C_2O_4^{2-}$ undergo shuttle phenomenon could also be explained by the Fourier transform infrared spectroscopy (FTIR) analysis of the electrolyte. By conducting FTIR measurements on the electrolyte of the ZnC_2O_4 • $2H_2O/LiFePO_4$ battery after 20 cycles, it is observed that the peaks of the electrolyte remain consistent with those of the initial CH_3COOLi electrolyte, without any additional peaks corresponding to $C_2O_4^{2-}$. This indicates that $C_2O_4^{2-}$ did not dissolve into the electrolyte, further confirming the absence of $C_2O_4^{2-}$ shuttle phenomenon.

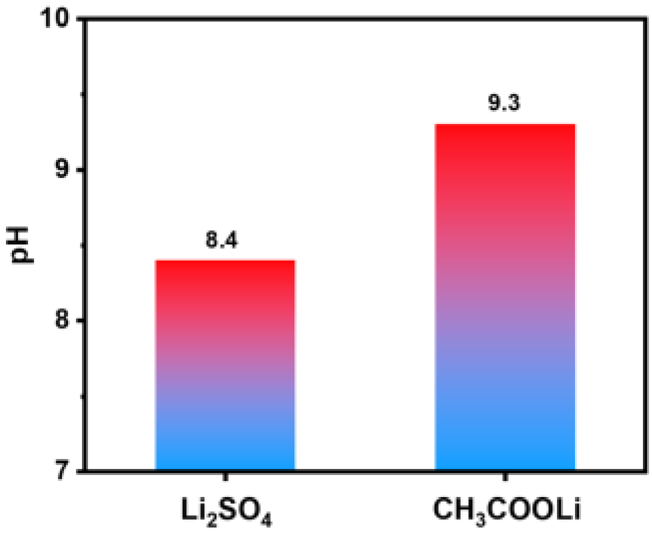

Figure S20. Comparison of pH between CH_3COOLi and Li_2SO_4 electrolyte.

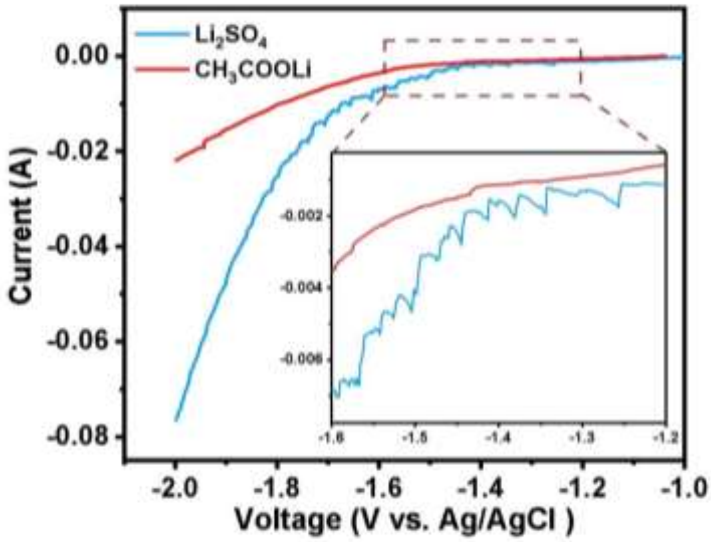

Figure S21. Comparison of LSV curves between CH_3COOLi and Li_2SO_4 electrolyte.

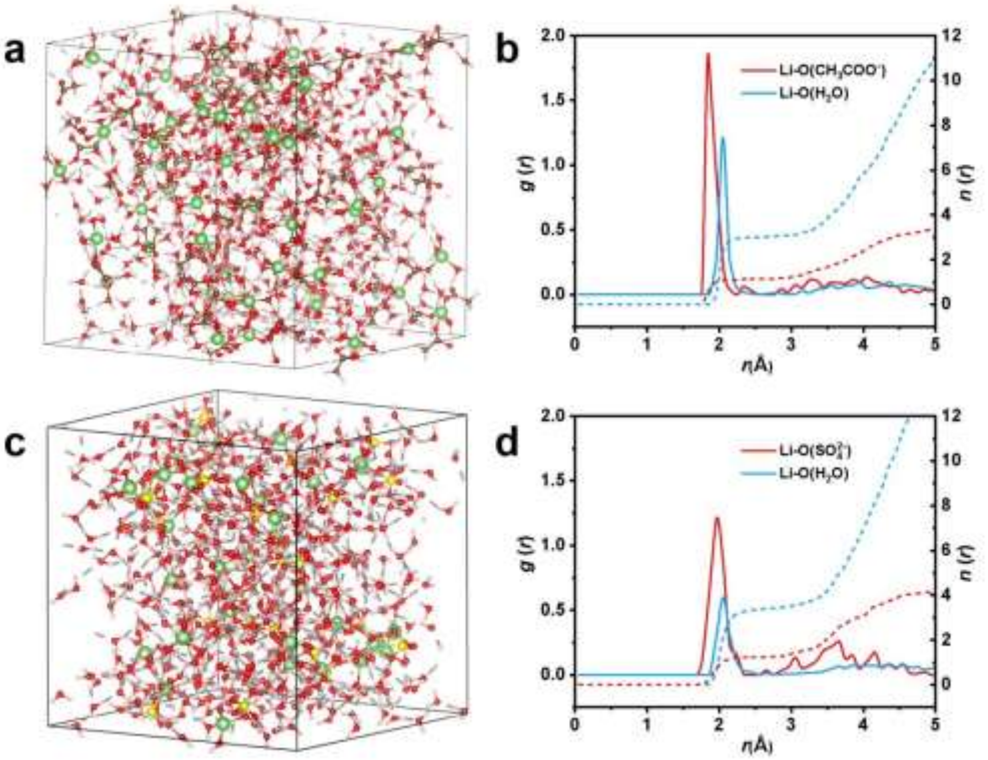

Figure S22. The MD simulation of different electrolyte. The snapshot and RDF image of (a-b) CH_3COOLi and (c-d) Li_2SO_4 electrolyte.

The solvation structure of the two electrolytes does not affect the choice of electrolyte. In these two electrolytes, the solvation structure of Li^+ only affects the interface reactions on the cathode side and is unrelated to the interface reactions on the anode side. Moreover, by comparing the radial distribution functions of the two electrolytes, it can be observed that they do not differ significantly. Therefore, the solvation structure of the two electrolytes does not affect the choice of electrolyte.

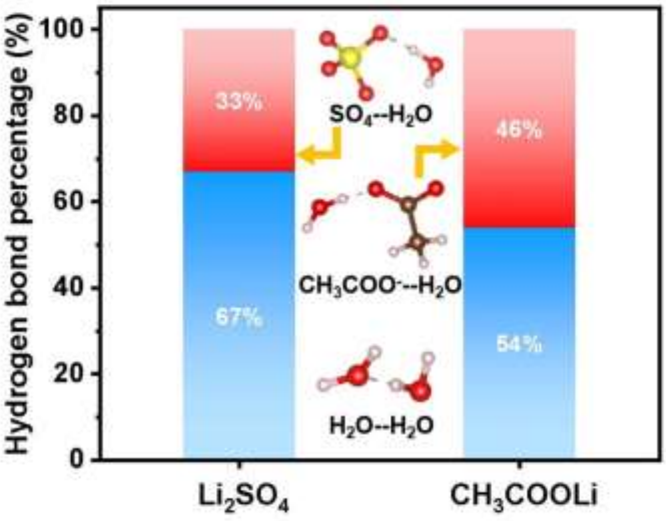

Figure S23. The percentage of hydrogen bonding in CH_3COOLi and Li_2SO_4 electrolyte.

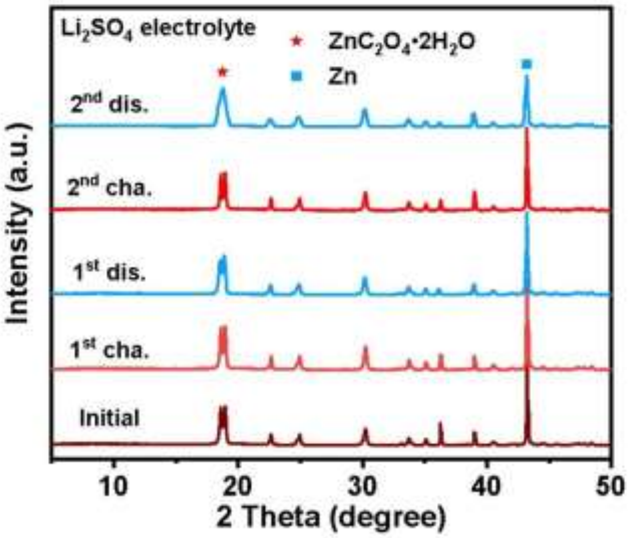

Figure S24. The *ex-situ* XRD pattern of $ZnC_2O_4{\bullet}2H_2O$ anode in the Li_2SO_4 electrolyte.

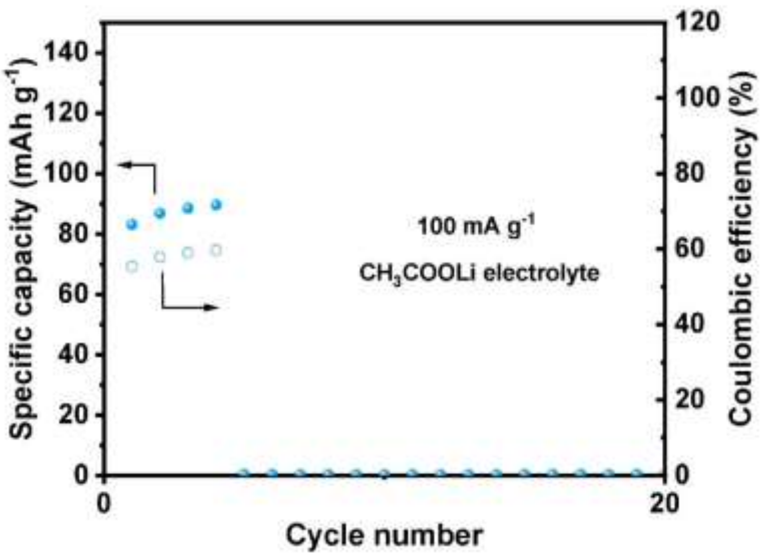

Figure S25. **The cycle performance of $LiMn_2O_4$ cathode with pure CH_3COOLi electrolyte.**

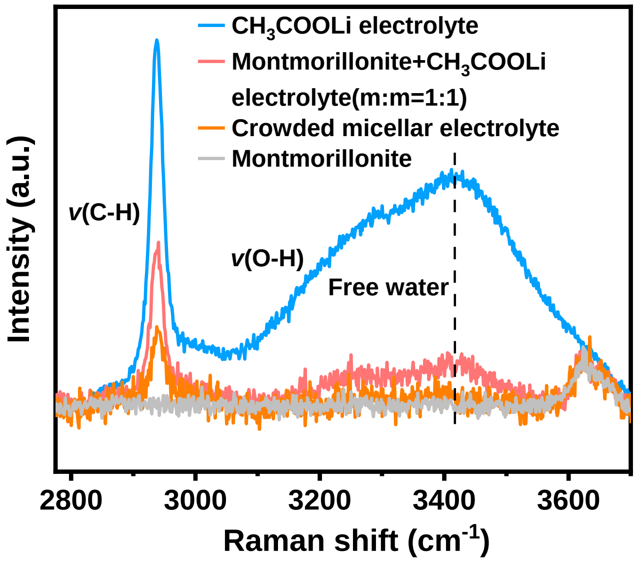

Figure S26. Effect of montmorillonite content on Raman spectra of electrolyte.

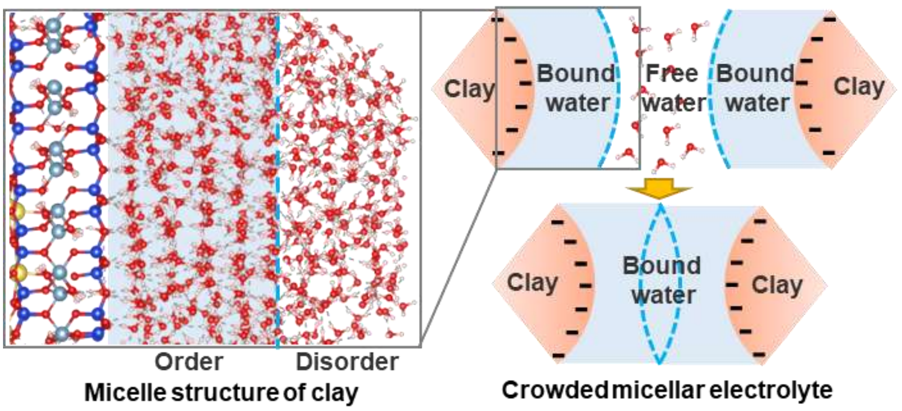

Figure S27. MD simulation of micelle structure of clay and the formation process of crowded micellar electrolyte.

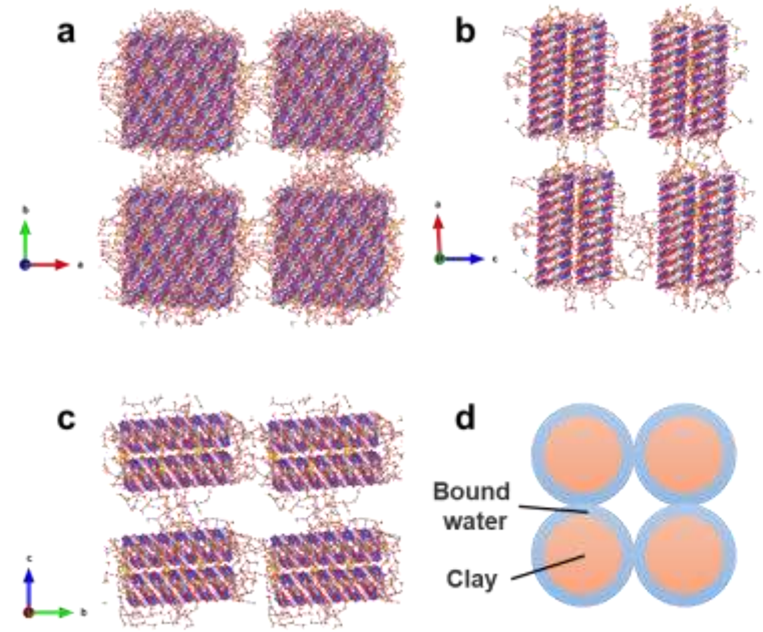

Figure S28. MD simulation of crowded micellar electrolyte. a-c, Snapshots of MD simulations from different perspectives. d, Structural schematic illustration of crowded micellar electrolyte.

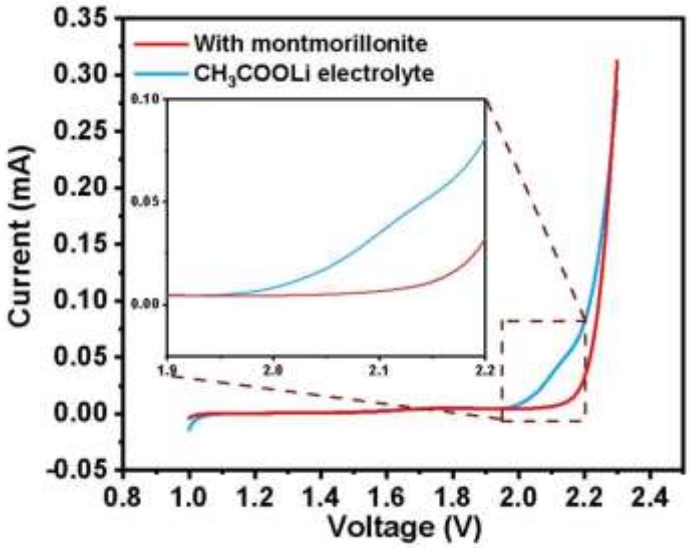

Figure S29. Comparison of LSV curves between pure CH_3COOLi electrolyte and crowded micellar electrolyte.

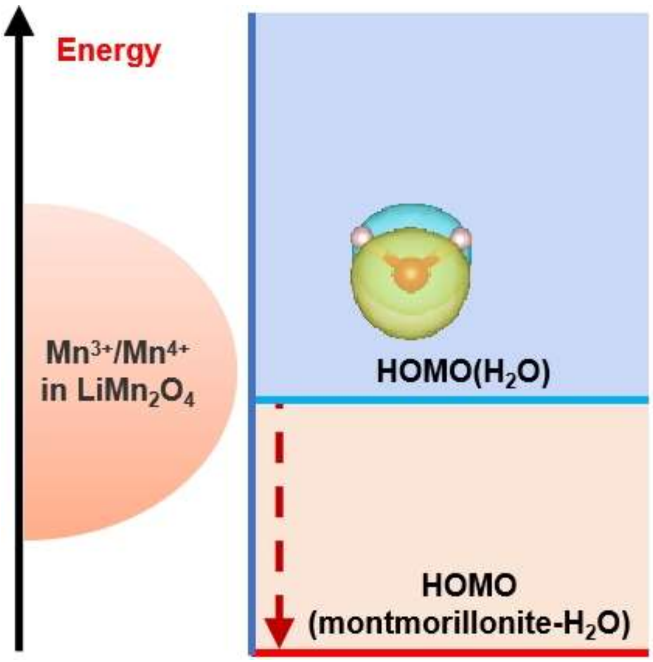

Figure S30. Energy level relationship of $LiMn_2O_4$ cathode with pure CH_3COOLi electrolyte and crowded micellar electrolyte.

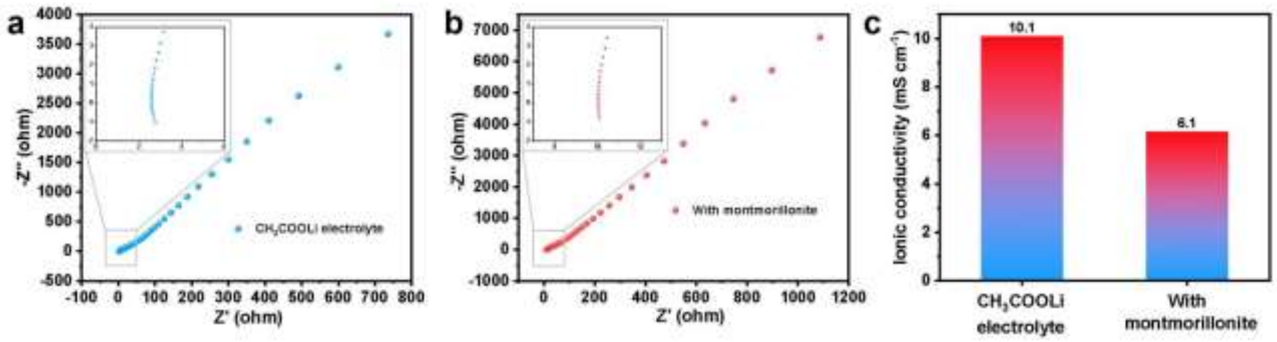

Figure S31. The comparison of ionic conductivity between pure CH_3COOLi electrolyte and crowded micellar electrolyte. The comparison of EIS with stainless steel - stainless steel in the (a) pure CH_3COOLi electrolyte and (b) crowded micellar electrolyte; (c) The corresponding comparison of ionic conductivity.

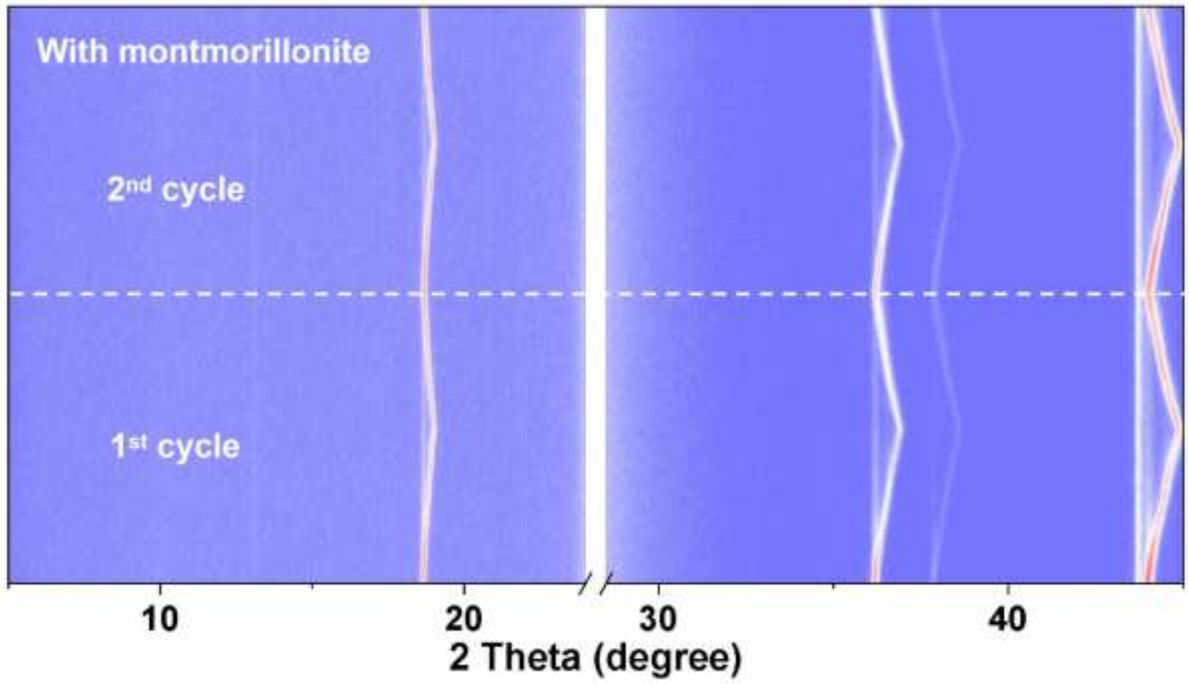

Figure S32. The *in-situ* XRD spectra of $LiMn_2O_4$ under crowded micellar electrolyte.

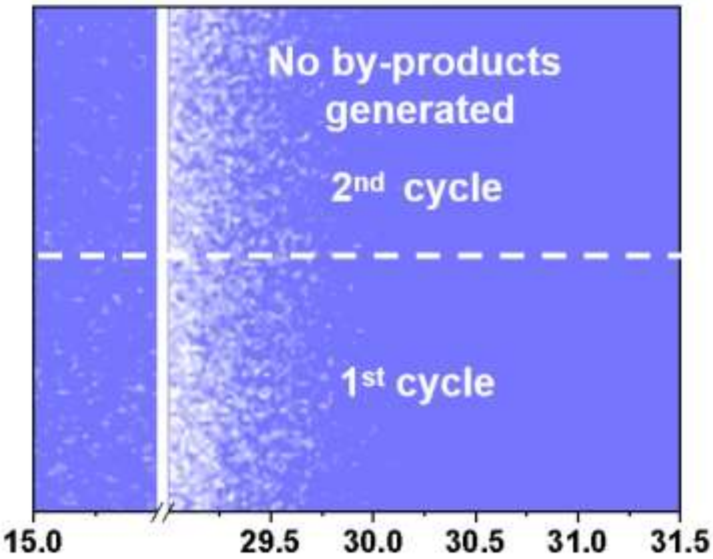

Figure S33. The partial enlarged *in-situ* XRD spectra of $LiMn_2O_4$ under crowded micellar electrolyte.

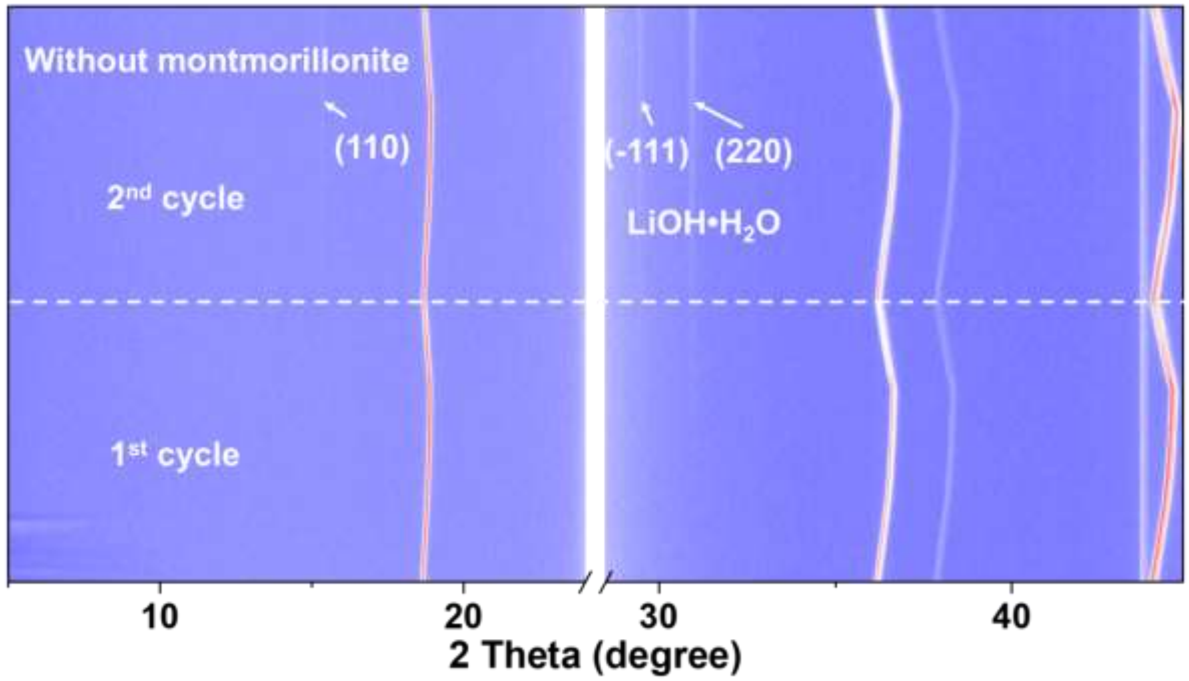

Figure S34. The *in-situ* XRD spectra of $LiMn_2O_4$ under pure CH_3COOLi electrolyte.

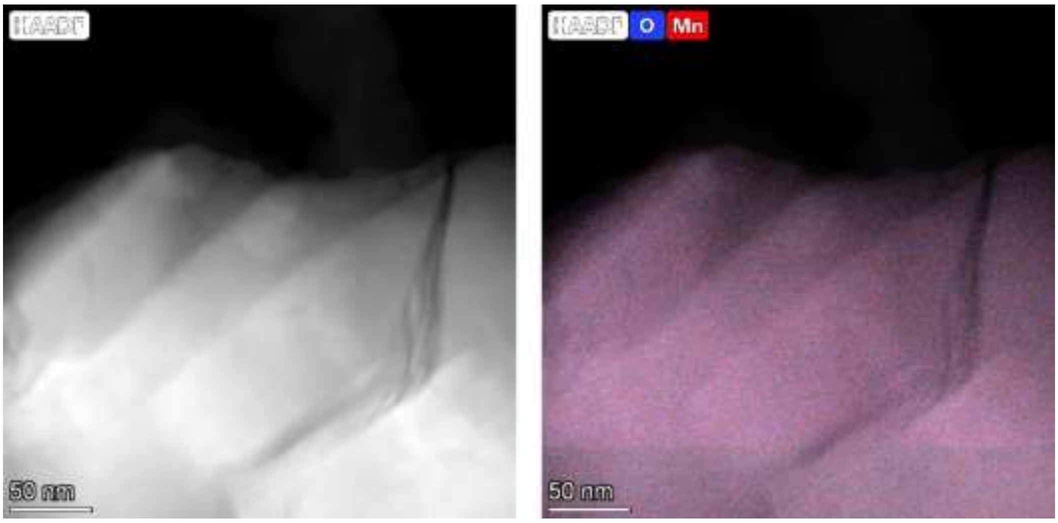

Figure S35. The STEM and EDS images of $LiMn_2O_4$ particle in the cracking region after 3 cycles at pure CH_3COOLi electrolyte.

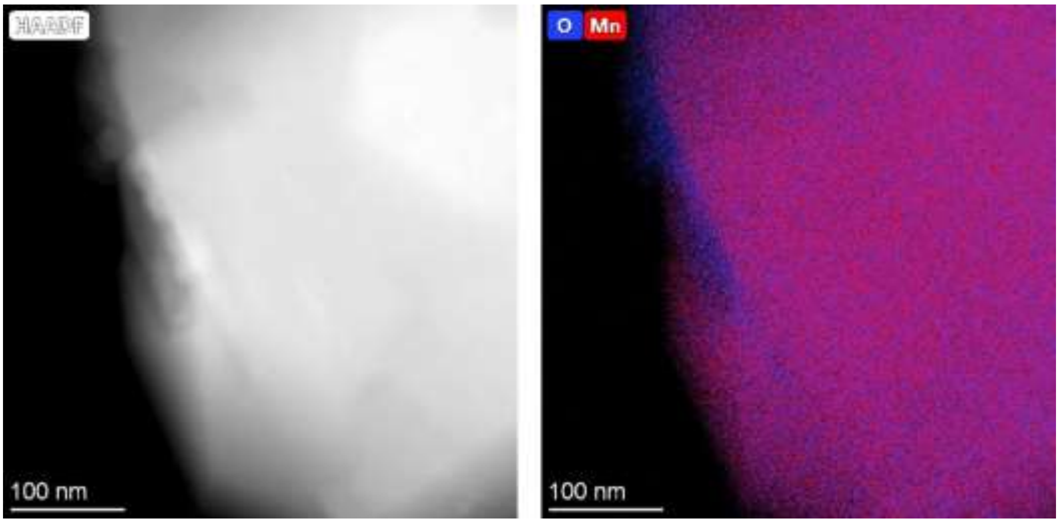

Figure S36. The STEM and EDS images of $LiMn_2O_4$ particle in the byproduct region after 3 cycles at pure CH_3COOLi electrolyte.

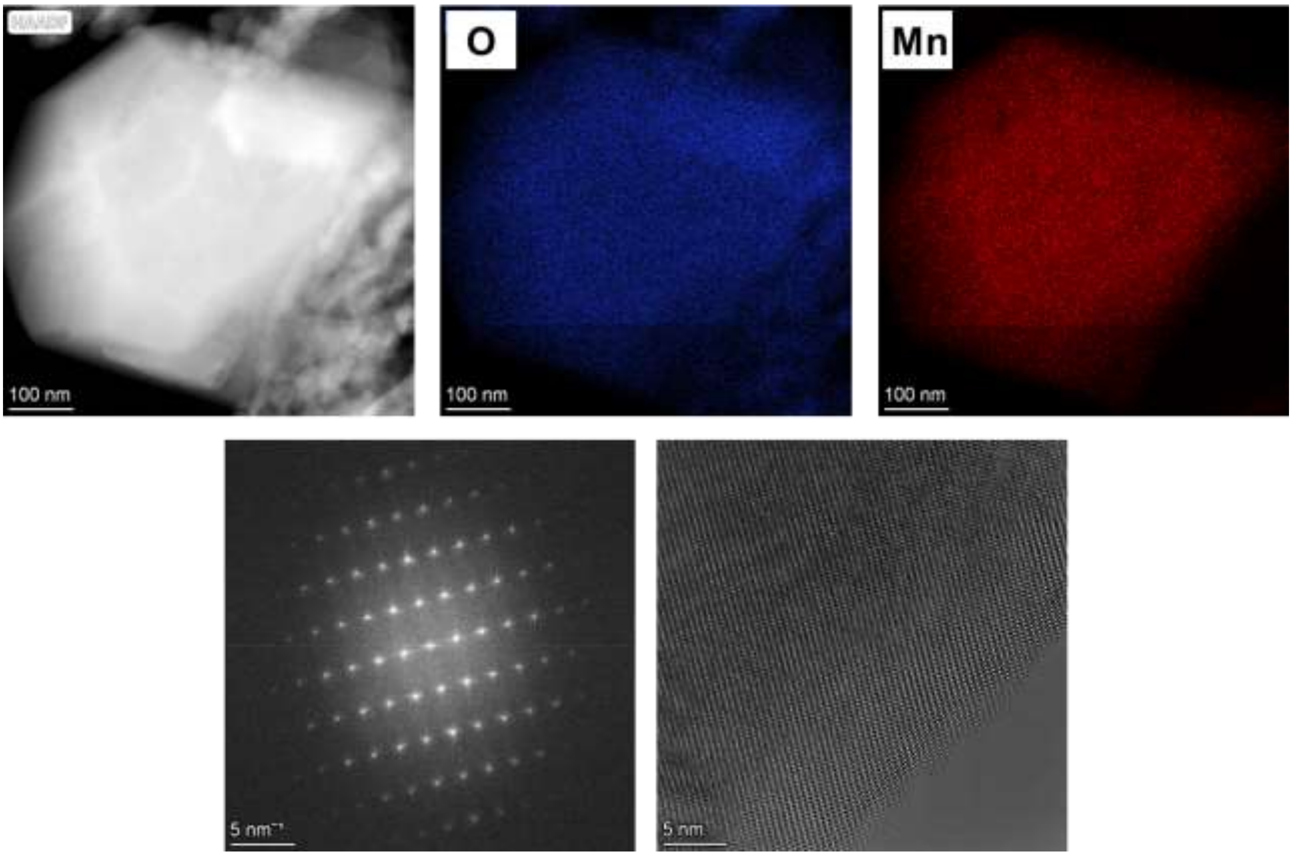

Figure S37. The STEM, EDS images, SAED pattern and HRTEM image of $LiMn_2O_4$ particle.

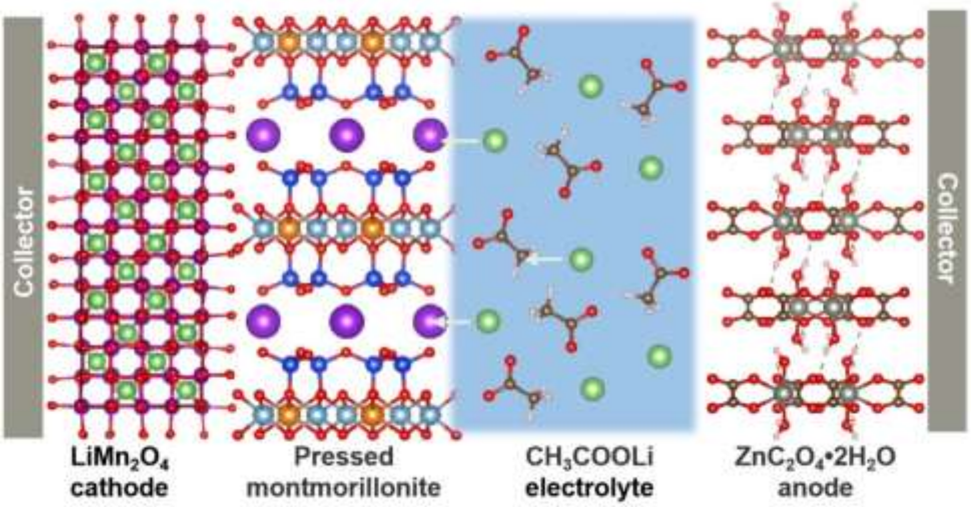

Figure S38. Schematic illustration of **designed $ZnC_2O_4 \cdot 2H_2O$ (anode) | CH_3COOLi (anodic electrolyte) | Montmorillonite (cathodic electrolyte) | $LiMn_2O_4$ (cathode).**

In the near-neutral high-voltage aqueous battery system designed in this work, the design of the asymmetric electrolyte is necessary due to the contradiction in the interface reaction between the anode and cathode. For the interface reaction at the anode, according to the compatibility principle between electrolyte and anode reactions, the LUMO energy level of the electrolyte needs to be higher than the chemical potential of the anode. Generally, the increase in the LUMO energy level of the electrolyte leads to the corresponding elevation of the HOMO energy level. However, for the high-voltage cathode interface, in order to match the cathode, the HOMO energy level of the electrolyte needs to be lower than the chemical potential of the cathode. Therefore, there exists a contradiction in the energy level requirements of the electrolyte at both interfaces. Additionally, for the conversion reaction to proceed normally, zinc ions need to first dissolve in the electrolyte. Thus, the electrolyte on the anode side requires an environment with high water content. However, the high water content on the cathode side may lead to manganese dissolution from the cathode, which is detrimental to stable cycling. Consequently, there are differences in the water content requirements of the electrolyte at both interfaces. These conflicting requirements make it impossible for a single electrolyte to simultaneously meet the needs of both the anode and cathode. Therefore, the design of asymmetric electrolytes is necessary.

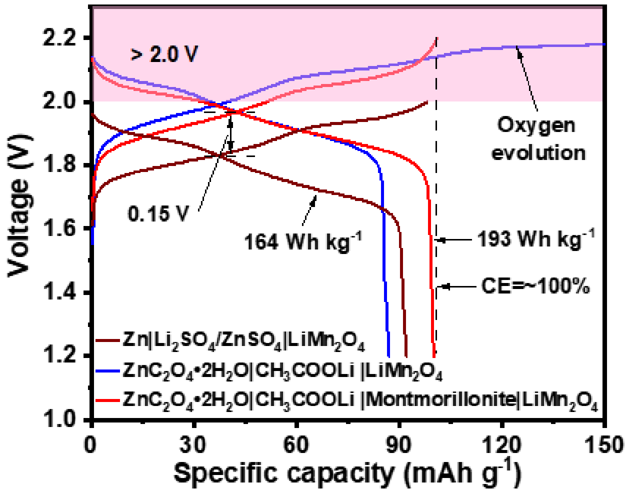

Figure S39. The effect of designed $ZnC_2O_4 \cdot 2H_2O$ (anode) | CH_3COOLi (anodic electrolyte) | Montmorillonite (cathodic electrolyte) | $LiMn_2O_4$ (cathode) on GCD curve.

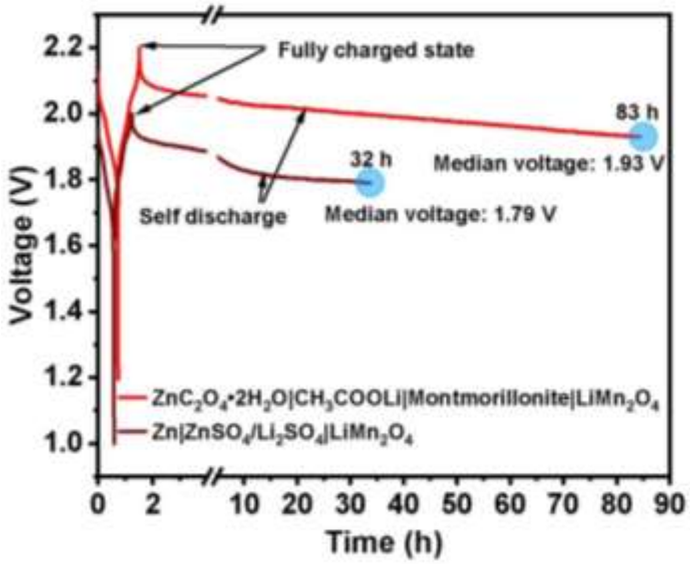

Figure S40. Comparison of self-discharge performance between the designed battery system and the ordinary $Zn/LiMn_2O_4$ system.

In order to demonstrate the self-discharge performance of the designed battery system, the self-discharge behavior of the conventional $Zn/LiMn_2O_4$ system is chosen for comparison. By charging both systems to the fully charged state and then allowing them to stand idle, the time taken for each system to self-discharge to their respective median voltage is observed. The designed battery system exhibits the relatively slow self-discharge process, with the self-discharge time of 83 h to the median voltage. In contrast, the comparative system demonstrates the notably faster self-discharge process, requiring only 32 h to reach the state of median voltage. Therefore, the designed battery system demonstrates better self-discharge performance compared to the

conventional Zn/$LiMn_2O_4$ system.

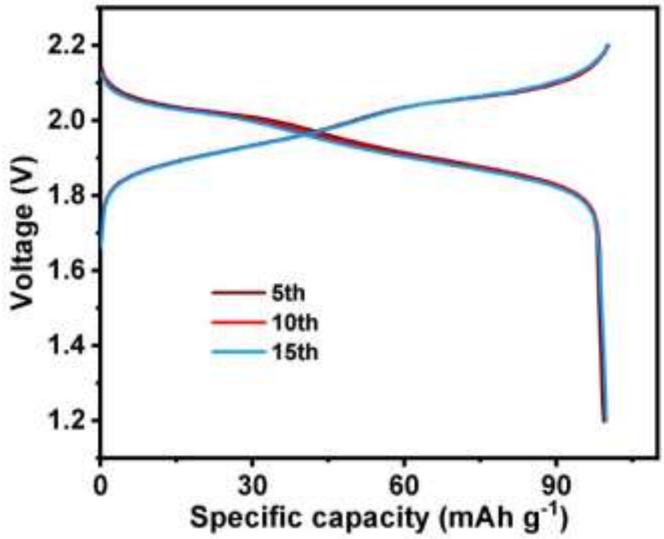

Figure S41. The GCD curves of designed ZnC_2O_4•$2H_2O$ (anode) | CH_3COOLi (anodic electrolyte) | Montmorillonite (cathodic electrolyte) | $LiMn_2O_4$ (cathode) at different cycles.

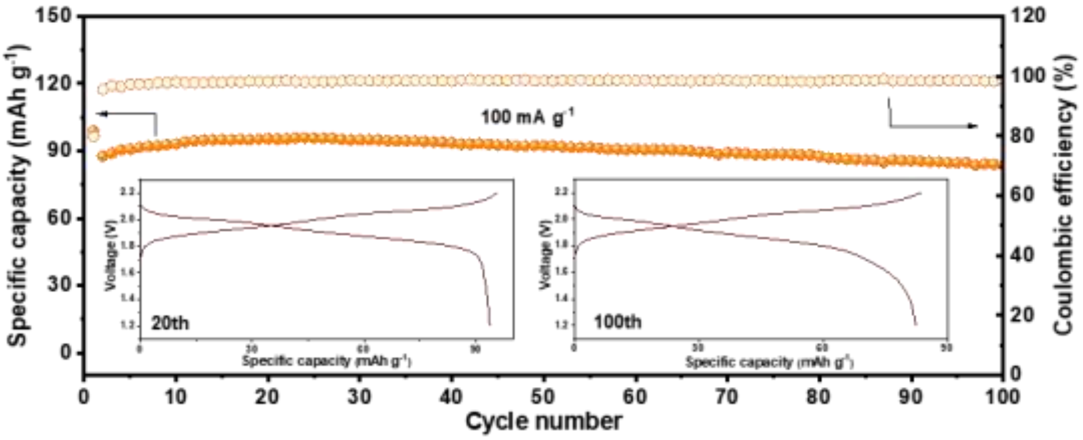

Figure S42. The cycle performance of designed system at 100 mA g^{-1} (inset: the GCD curves for the 20th and 100th cycles).

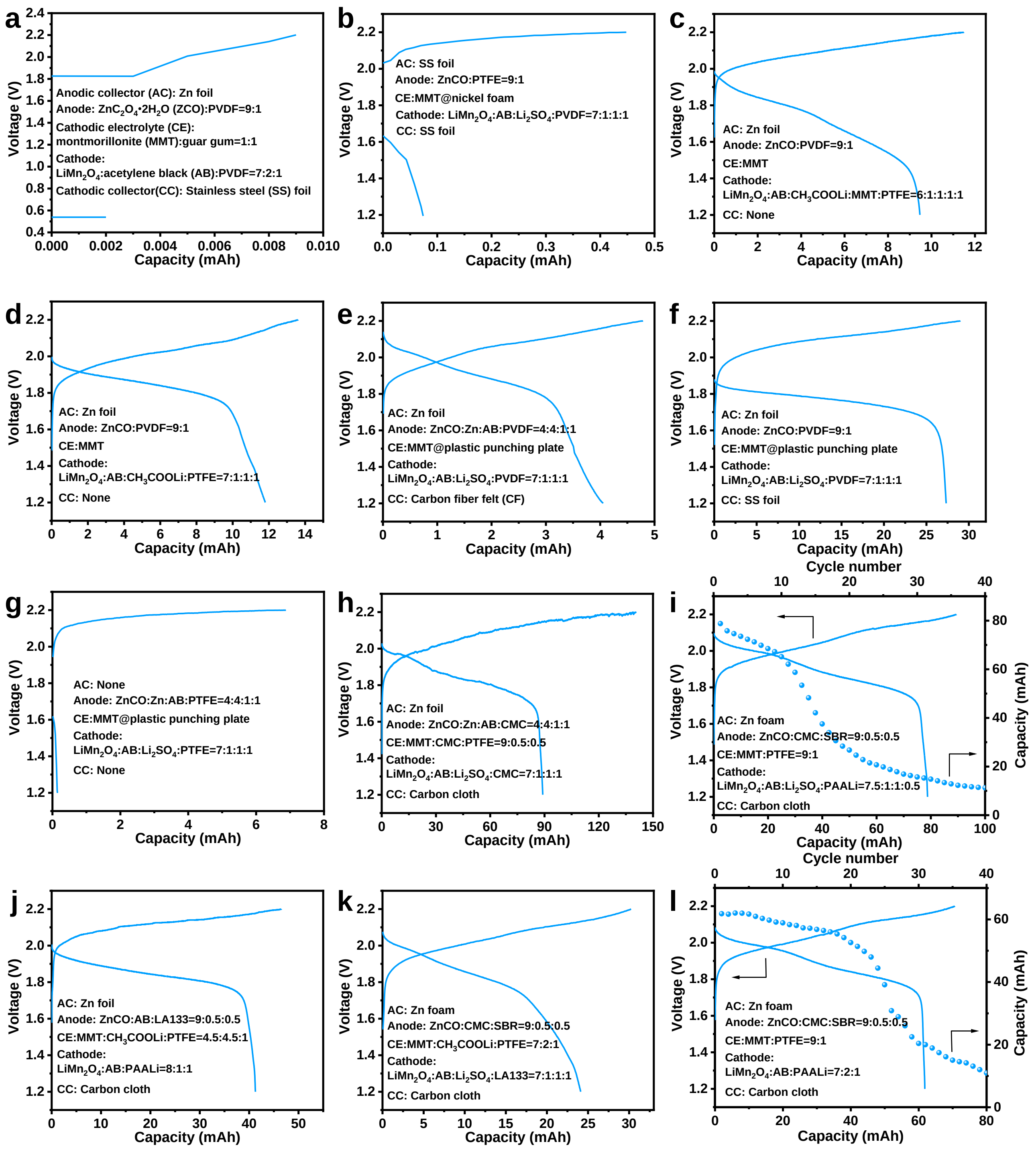

Figure S43. The regulatory effects of ten factors on the performance of pouch cell. a, The effect of the same assembly method employed in the coin cell on the GCD curve of pouch cell (Anodic collector (AC): Zn foil, Anode: $ZnC_2O_4 \cdot 2H_2O$ (ZCO):PVDF=9:1, Cathodic electrolyte (CE): montmorillonite (MMT):guar gum=1:1, Cathode: $LiMn_2O_4$: acetylene black (AB) : PVDF = 7:2:1, Cathodic collector (CC): Stainless steel (SS) foil). **b-l**, The influence by adjusting ten factors on the performance of pouch cell (**b,** AC: SS foil, Anode: ZnCO:PTFE=9:1, CE:MMT@nickel

foam, Cathode: $LiMn_2O_4$:AB:Li_2SO_4:PVDF=7:1:1:1, CC: SS foil; **c,** AC: Zn foil, Anode: ZnCO : =9:1, CE:MMT, Cathode: $LiMn_2O_4$:AB:CH_3COOLi:MMT:PTFE=6:1:1:1:1, CC: None; **d,** AC: Zn foil, Anode: ZnCO:PVDF=9:1, CE: MMT, Cathode: $LiMn_2O_4$:AB:CH_3COOLi:PTFE=7:1:1:1, CC: None; **e,** AC: Zn foil, Anode: ZnCO:Zn:AB:PVDF=4:4:1:1, CE:MMT@plastic punching plate, Cathode: $LiMn_2O_4$:AB:Li_2SO_4:PVDF=7:1:1:1, CC: Carbon fiber felt (CF); **f,** AC: Zn foil, Anode: ZnCO:PVDF=9:1, CE:MMT@plastic punching plate, Cathode: $LiMn_2O_4$: AB : Li_2SO_4 : PVDF = 7:1:1:1, CC: SS foil; **g,** AC: None, Anode: ZnCO:Zn:AB:PTFE=4:4:1:1, CE:MMT@plastic punching plate, Cathode: $LiMn_2O_4$:AB:Li_2SO_4:PTFE=7:1:1:1, CC: None; **h,** AC: Zn foil, Anode: ZnCO:Zn:AB:CMC=4:4:1:1, CE: MMT:CMC:PTFE=9:0.5:0.5, Cathode: $LiMn_2O_4$: AB : Li_2SO_4 : CMC = 7:1:1:1, CC: Carbon cloth; **i,** AC: Zn foam, Anode: ZnCO:CMC:SBR=9:0.5:0.5, CE: MMT:PTFE=9:1, Cathode: $LiMn_2O_4$:AB:Li_2SO_4:PAALi=7.5:1:1:0.5, CC: Carbon cloth; **j,** AC: Zn foil, Anode: ZnCO:AB:LA133=9:0.5:0.5, CE: MMT:CH_3COOLi:PTFE=4.5:4.5:1, Cathode: $LiMn_2O_4$: AB : PAALi =8:1:1, CC: Carbon cloth; **k,** AC: Zn foam, Anode: ZnCO : CMC : SBR = 9:0.5:0.5, CE: MMT:CH_3COOLi:PTFE=7:2:1, Cathode: $LiMn_2O_4$:AB:Li_2SO_4:LA133=7:1:1:1, CC: Carbon cloth; **l,** AC: Zn foam, Anode: ZnCO:CMC:SBR=9:0.5:0.5, CE:MMT:PTFE=9:1, Cathode: $LiMn_2O_4$:AB:PAALi=7:2:1, CC: Carbon cloth).

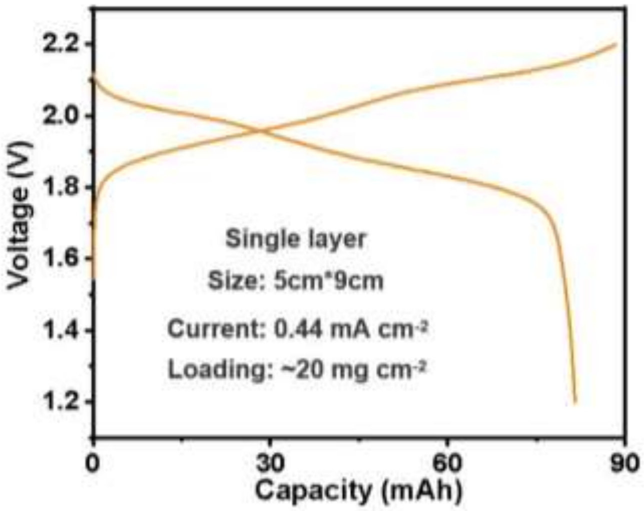

Figure S44. The GCD curve of single layer pouch cell with the size of 5cm×9cm at 0.44 mA cm^{-2}.

Currently, most pouch cells utilize vanadium-based cathodes, which typically have the low voltage of only around 0.6V. The pouch cell used in our work could obtain the voltage platform above 2V, which offers a significant advantage in voltage compared to other batteries. Additionally,

the pouch cell used in this work can effectively utilize the capacity of battery. By increasing the electrode loading from 0.6 mg cm^{-2} to 20 mg cm^{-2}, the capacity retention could reach 86%, which is particularly challenging in other AZIBs. Through the comprehensive analysis above, it is evident that the pouch cell used in this work holds advantages in both voltage and capacity compared to other batteries, thereby demonstrating the overall advantage in energy density. As for energy efficiency, the pouch cell used in our work benefits from the three-dimensional structure in the anode and the incorporation of mobile transport ions in the cathode. This enables the pouch cell devices at the Ah level to exhibit similar GCD curves and polarization voltage compared with coin cells. This characteristic is uncommon in other AZIBs. Typically, the GCD curves of pouch cells in other AZIBs differ significantly from coin cells, especially in terms of polarization voltage, where pouch cells tend to exhibit greater polarization compared to coin cells. This disparity often leads to significantly higher energy consumption during the charge process than the energy release during the discharge process, resulting in substantial energy waste and low energy efficiency. However, in our work, the polarization issue has been effectively addressed, resulting in significant advantages in energy efficiency compared to other AZIBs.

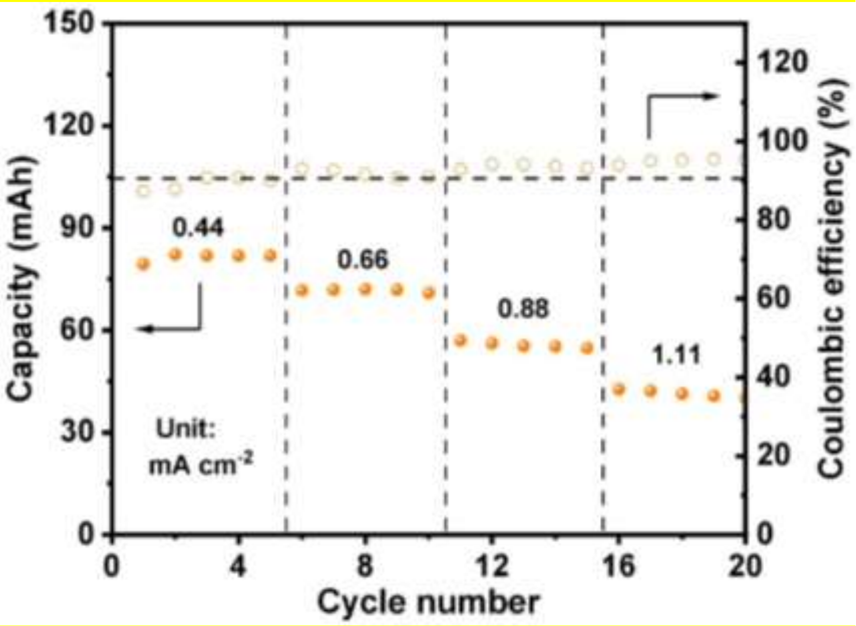

Figure S45. The rate performance of the single-layer pouch cell with dimensions of 5cm × 9cm.

Under current densities of 0.44 mA cm^{-2}, 0.66 mA cm^{-2}, 0.88 mA cm^{-2}, and 1.11 mA cm^{-2}, the pouch cell could achieve the capacities of 82 mAh, 72 mAh, 55 mAh, and 42 mAh, respectively.

And corresponding coulombic efficiencies are 90%, 91%, 94%, and 95%, respectively. It is evident that the pouch cell designed in this work, utilizing the three-dimensional anode structure, significantly improves the sluggish kinetics of the anodic conversion reaction, thereby achieving excellent rate capability. However, the hydrogen evolution side reaction intensifies with the scaling up of the battery system, particularly under the test conditions of low current density, where the battery only exhibits the coulombic efficiency of 90%. As the current density increases, the hydrogen evolution side reaction relatively diminishes, leading to the improvement in Coulombic efficiency with increasing current density. The hydrogen evolution side reaction can lead to capacity degradation in pouch cell. Therefore, further in-depth research is still required to improve the performance of pouch cell.
