# Supplementary material for: Conversion-type anode chemistry with interfacial compatibility toward Ah-level near-neutral high-voltage zinc ion batteries
Source: Natl Sci Rev. 2024 May 25;11(7):nwae181. doi: 10.1093/nsr/nwae181 (PMC11193386; doi:10.1093/nsr/nwae181)
Supplement: nwae181_Supplemental_Files [file nwae181_supplemental_files.zip › Teaser text.docx]

This paper proposes the conversion-type anode chemistry and interfacial compatibility to design Ah-level near-neutral high-voltage zinc ion batteries.
